# Healthcare utilization and costs for patients with Parkinson’s disease in Taiwan

**DOI:** 10.1186/s12883-024-03988-3

**Published:** 2025-01-03

**Authors:** Kuan-Chen Chen, Li-Jung Elizabeth Ku, Ya-Hui Hu, Yu Sun, Alexis Elbaz, Pei-Chen Lee

**Affiliations:** 1 Department of Health Care Management, National Taipei University of Nursing and Health Sciences, No. 365, Mingde Road, Peitou District, Taipei City, 112 Taiwan; 2 Department of Public Health, College of Medicine, National Cheng Kung University, No.1, University Road, Tainan City, 701 Taiwan; 3 Department of Neurology, En Chu Kong Hospital, No. 399, Fuxing Road, Sanxia District, New Taipei City, 237 Taiwan; 4 Université Paris-Saclay, UVSQ, Gustave Roussy, Inserm, Team «Exposome, Heredity, Cancer, and Health», CESP UMR 1018, Villejuif, F-94807 France

**Keywords:** Parkinson’s disease, Healthcare utilization, Cost, Economic burden, National Health Insurance

## Abstract

**Background:**

Parkinson’s disease (PD) exerts a considerable burden on the elderly. Studies on long-term costs for Parkinson’s disease patients in Taiwan are not available.

**Objectives:**

This study aims to examine the medical resource utilization and medical costs including drug costs for PD patients in Taiwan over up to 15 years of follow-up.

**Methods:**

Incident PD patients and matched non-PD subjects were identified between 2003 and 2016 from the National Health Insurance (NHI) research database. Differences in annual healthcare utilization and costs between PD and non-PD subjects from 2003 to 2018 were predicted by generalized linear models. We performed analyses stratified by PD severity and also by age, gender, and duration of follow-up.

**Results:**

We identified 50,290 PD cases and 201,153 non-PD subjects. From the payer’s perspective, the average total medical costs (drug costs) associated with PD and non-PD subjects were NT$631,080 (NT$222,810) and NT$480,880 (NT$140,120), respectively. Total medical and drug costs of PD after diagnosis remained high, from NT$138,487 per patient in the first year following diagnosis up to NT$154,676 per patient at year 15. The largest components of costs were for outpatient care (55% of total medical costs), and total drugs cost (35% of total medical costs). Patients with severe PD incurred higher total medical costs compared to those with moderate or mild PD, with outpatient and inpatient costs as well as drug costs rising with disease severity.

**Conclusions:**

This is the first study of its kind in Taiwan that comprehensively analyzes long-term healthcare utilization and costs among PD patients. PD imposes a significant economic burden in Taiwan, with medical (drug) costs being 1.31 (1.59) times that of non-PD individuals and costs increasing substantially with PD severity. Our findings can aid health policymakers in understanding the healthcare needs and medical costs of PD patients, supporting effective policy formulation.

**Supplementary Information:**

The online version contains supplementary material available at 10.1186/s12883-024-03988-3.

## Introduction

The World Health Organization (WHO) predicts that by 2040, neurodegenerative diseases, including Parkinson’s disease (PD), will become the second most common cause of death after cardiovascular disease [1]. A recent US study estimated a prevalence of approximately one million individuals with diagnosed PD in 2017 and a total economic burden of $51.9 billion. By 2037, PD prevalence in the US is projected to exceed 1.6 million and the total economic burden is projected to exceed $79.1 billion [2]. By 2025, Taiwan’s elderly population (aged 65 and above) is expected to reach approximately 20%, alongside a projected increase in healthcare expenditure, particularly for age-related conditions such as PD. As the proportion of older people in the Taiwanese population has grown, PD incidence and prevalence rates have showed marked increasing trends [3]. This underscores the pressing healthcare burden that PD poses in Taiwan, highlighting the need to understand the local context to address better the disease’s impact on Taiwan’s healthcare system.

Previous studies showed that PD has a large societal and financial burden due to its progressive and disabling neurodegenerative disorder [2, 4–9]. While prior studies investigated healthcare costs associated with PD, their reliance on cross-sectional data and short follow-up periods inherently constrained their capacity to capture the cumulative and progressive financial impact associated with disease progression [8–10]. Most previous PD medical utilization studies relied on patients’ self-reports of healthcare usage and were usually conducted over short follow-up periods, which does not allow calculation of annual changes in healthcare utilization as the disease progresses. Specifically, these methodologies often overlook stratification by disease severity, which can lead to an underestimation of the economic burden in the advanced stages of PD. This limitation highlights a significant gap in the literature, as the lack of longitudinal analyses restricts our understanding of the comprehensive extent to which healthcare costs escalate as disease severity progresses over time.

​Additionally, previous studies examined the economic burden of PD from the perspective of total medical costs and did not specifically estimate drug costs. Disease progression is associated with increased morbidity, fluctuations in symptom control, impairments in activities of daily living, and mortality, resulting in an impressive cumulative medical cost associated with hospitalization and medical services [11, 12]. Consequently, healthcare utilization patterns among PD patients change substantially as the disease advances.

Given the paucity of data on medical utilization of PD in Asian populations, we aimed to estimate the real-world healthcare use and long-run cost of PD in Taiwan. The scarcity of data on medical utilization in Asian populations, including Taiwan, highlights the importance of this study, as healthcare delivery systems in Asia differ from those in Western countries. For instance, Taiwan’s National Health Insurance (NHI) provides universal coverage with low out-of-pocket costs, potentially influencing healthcare utilization patterns for PD. The primary purpose of this study is to estimate the medical resources utilization and medical costs, including drug costs, of incident PD patients over up to 15 years of follow-up, and to compare them to that of persons without PD. A secondary purpose is to evaluate the economic burden related to different degrees of severity of PD. The study focuses on how much new patients will cost as the disease progresses. These data would be helpful for healthcare providers and policy makers as they would provide a more complete picture of the economic burden of patients with PD and would facilitate the formulation of healthcare policy plans in Asian countries.

## Methods

### Data sources

In this population-based cohort study, we accessed all the data at the Health and Welfare Data Science Center (HWDC) under the Ministry of Health and Welfare, including the National Health Insurance Research Database (NHIRD) and mortality databases between 2000 and 2018. Taiwan launched a single-payer NHI plan on March 1, 1995, and developed the NHI computerized reimbursement database, i.e., NHIRD. The NHI is a compulsory program for all citizens and legal residents. NHIRD contains nationwide health claims data, including inpatient and outpatient visits and prescription information, for all forms of healthcare services for 99% of the Taiwanese population [13]. Taiwan's NHIRD is a source of population-level data that generates real-world evidence to support clinical decision-making and healthcare policy development. Regarding drug pricing, drug prices are reasonably adjusted based on market transaction conditions by the government. Based on the National health claims data, we selected PD incident cases diagnosed between 2003 and 2016 and persons without PD, and compared the healthcare utilization (including outpatient, inpatient, accidents and emergency care utilization and related costs) among these two groups up to 2018. We also linked the NHIRD with death registry via the unique and anonymous personal identification numbers to obtain the person-level information. The study was approved by the Institutional Review Board of the Taiwan Adventist Hospital (110-E-02) and the National Cheng Kung University Human Research Ethics Committee (NCKU HREC-E-111-49-2).

### Participants

We identified PD patients in the NHIRD as those with a PD code according to the International Classification of Disease (9th Revision, Clinical Modification, ICD-9-CM, code 332.0; ICD-10-CM code G20) and at least three medical claims (either ambulatory or inpatient care) between 2000 and 2018 [7, 10, 12]. We defined the date of the first medical record for PD after 2003 as the index date, and we identified incident cases after 2003 by examining data since 2000 to exclude prevalent cases. Several criteria were previously validated to identify incident PD cases [14]. Briefly, we required PD cases to have at least two PD diagnosis and two claims of any anti-parkinsonism medication (Anatomical Therapeutic Chemical, ATC, codes: C04AE51, G02CB01, G02CB02, G02CB03, N04BA01, N04BA02, N04BA03, N04BB01, N04BC02, N04BC04, N04BC05, N04BC07, N04BC09, N04BD01, N04BD02, N04BX02, V03AB91) at least 90 days apart, and at least three claims of any anti-parkinsonism medication within 2 years after the index date.

To ensure the validity of the PD diagnosis, we further excluded patients (1) younger than 35 years old at the index date, (2) with codes for secondary PD (ICD-9-CM code: 332.1; ICD-10-CM code: G21) at baseline, (3) with neuroleptic medications within 180 days before the index date, (4) three or more medical claims with a dementia diagnosis code before the index date to avoid misdiagnosis with other conditions (e.g., dementia with Lewy bodies, other causes of dementia with parkinsonism), and (5) with codes for progressive supranuclear palsy (ICD-9-CM code: 333.0; ICD-10-CM code: G23.1), multiple system atrophy (ICD-9-CM code: 333.0; ICD-10-CM code: G23.2, G23.3), and corticobasal degeneration (ICD-9-CM code: 331.6; ICD-10-CM code: G31.85) after the index date. Given that the number of patients who received deep brain stimulation (DBS) during the study period was very small (*N* = 190, 0.4%), removing them from the analysis did not alter the results. Patients were censored either at the date of death or loss of insurance status. There were no missing data for the variables that we analyzed.

To select a comparison cohort, we first excluded subjects who had been previously diagnosed with PD or dementia before 2003. We then used risk-set sampling (4:1 ratio) to identify non-PD subjects who were matched to PD cases on age, gender, year of PD diagnosis, and city/county of residence at the index date. The final sample included 251,433 participants (50,290 PD cases, 201,153 non-PD subjects; Appendix Figure 1). Members of the comparison cohort who later developed PD (< 3%) were censored at the diagnosis date and then included as PD cases. We assigned the same index date as the case to each matched non-PD subject.

PD cases were further classified into mild (*n* = 16,595), moderate (*n* = 16,597), and severe PD (*n* = 17,098) based on the distribution of the annual cumulative levodopa equivalent dose for levodopa, dopamine agonists, and other PD medications (< 33rd percentile, 33rd– < 66th percentile, ≥ 66th percentile, respectively) in the three years after the index date. This classification is considered as a valid proxy for clinical severity and was used to grade PD severity in previous studies [12].

### Definitions of variables

Our main outcome variables were the frequency and costs of care, including outpatient, inpatient and accidents (i.e., inpatient and outpatient emergency care). As PD patients are prone to falls due to gait disturbances, we specifically assessed accidents and emergency care in outpatient and inpatient care. In addition, we also estimated drug costs under different medical services.

All costs were adjusted first by the global budget system (GB) point value reimbursed according to Bureau of National Health Insurance in each quarter of each year [15] and next by the Consumer Price Index (CPI) (baseline 2016), called adjusted values. Participants’ baseline characteristics (i.e., at the index date) were age, gender, insurance premium, urbanization status, and the Charlson Comorbidity Index (CCI), a widely used indicator of medical comorbidities [16]. Insurance premium and urbanization level were used as proxy measures of socioeconomic position. Insurance premium was based on enrollees’ monthly insurance payment: $0 (insured as a dependent), less than median (NT$19,200), and greater than median (NT$19,200). Urbanization status was categorized into three groups: urban, satellite city/town, and rural area based on both total population and density criteria [17]. CCI was based on ICD codes for the period within one year before the index date categorized into three groups (0, 1, ≥ 2).

### Statistical analyses

We followed PD and non-PD subjects from the index date to the end of 2018, loss of their beneficiary status, or death, whichever occurred first. We used Pearson’s χ2 test to examine socio-demographic characteristics differences between PD and non-PD subjects. We also separated patients according to their vital status at the end of the follow-up, and further analyzed the sociodemographic characteristics of deceased and surviving PD participants.

In our analyses, duration refers to the length of study observation, with patients being followed from their index date until the end of 2018, loss of insurance status, or death, whichever came first. This setup ensures each patient’s data accurately reflects their time in the study, which mitigates potential survivor bias. By analyzing the data based on the duration of follow-up (e.g., 0–4 years, 5–9 years, and ≥ 10 years), the study captures the incremental costs associated with extended observation periods, addressing the influence of varying follow-up times on cost and utilization estimates.

Predicted values of medical care utilization and costs between PD and non-PD subjects were assessed through a generalized regression model with an identity link for continuous outcomes (e.g., linear regression), adjusted for age, gender, urbanization status, insurance premium, and CCI. All dependent variables were chosen a priori and not based on statistical significance; nevertheless, they were all significantly associated with medical care utilization and costs. There was no evidence of multicollinearity as the variation inflation factor was always < 5. Paired *T*-tests were used to compare differences in regression-predicted frequencies and costs of care between PD cases and non-PD subjects. All predicted values were also compared according to the severity of PD. As dementia is a major comorbidity of PD that may lead to different patterns of medical costs and drug costs, we also compared medical and drug costs in PD cases with and without dementia.

For predicted values of medical care utilization and costs between PD and non-PD subjects, we also conducted analyses stratified by mean age at the index date (< 72 years, ≥ 72 years), gender (female, male) and disease duration (0–4 years, 5–9 years, ≥ 10 years). Similarly, when we compared predicted values of medical care utilization and costs according to the severity of PD and vital status, we repeated analyses stratified by age, gender, and disease duration.

## Results

We included 50,290 incident PD patients and 201,153 non-PD subjects (49% females; mean age = 7.4 years, SD = 10.1; Table 1). PD cases were less likely to live in satellite cities/towns, and more likely to have a CCI score greater than 1 compared to non-PD subjects. The average length of follow-up was 5.9 (SD = 3.5) years for PD patients and 6.6 (SD = 3.8) years for non-PD subjects. During the study period, the mortality rate among PD patients and non-PD subjects were 6.87/100 person-years and 4.61/100 person-years, respectively. PD patients with higher insurance premiums, typically associated with higher socioeconomic status, showed more frequent access to outpatient care and incurred higher medication costs. Similarly, patients residing in urban areas showed higher utilization rates than those in rural areas, likely due to greater access to healthcare resources.

**Table 1:** Sociodemographic characteristics of patients with PD and non-PD

Variables	PD patients (*N* = 50,290)	Non-PD (*N* = 201,153)	*P-value*^1^
	n (%)	n (%)	
Age (years)			1.00
< 70	16,926 (33.7)	67,704 (33.7)	
70–74	9,661 (19.2)	38,644 (19.2)	
75–79	11,002 (21.9)	44,006 (21.9)	
≧80	12,701 (25.3)	50,799 (25.3)	
Mean (SD)	72.4 (10.1)	72.4 (10.1)	
Gender			1.00
Female	24,436 (48.6)	97,742 (48.6)	
Male	25,854 (51.4)	103,411 (51.4)	
Insurance premium (NT$)^a^			0.16
Dependent	31,664 (63.0)	127,345 (63.3)	
< Median (19,200)	5,772 (11.5)	23,230 (11.6)	
≧Median	12,854 (25.6)	50,578 (25.1)	
Mean (SD)^a^	17,750.0 (18,302.0)	17,937.0 (18,247.0)	
Urbanization status			0.04
Urban	26,848 (53.4)	106,289 (52.8)	
Satellite city/town	16,532 (32.9)	67,324 (33.5)	
Rural area	6,910 (13.7)	27,540 (13.7)	
Charlson Comorbidity Index^b^			< .001
0	25,918 (51.5)	127,923 (63.6)	
1	13,302 (26.5)	39,794 (19.8)	
≥ 2	11,070 (22.0)	33,436 (16.6)	

*SD* Standard deviation

^a^NT$ = New Taiwan Dollars

^b^Within one year before the index date

^1^χ2 test

Table 2 presents the predicted values of medical care utilization, cost of care, and drug costs for PD patients compared to non-PD subjects. PD patients had a higher mean predicted frequency of all types of care compared with non-PD subjects (Table 2). PD cases incurred 1.31 times higher total medical costs (predicted values: NT$631,080 vs. NT$480,880; *p* < 0.0001) than non-PD cases. Costs of outpatient care, inpatient care, and accidents were all higher in PD patients compared to their counterparts. Across different types of health services, outpatient care represents the largest part of total medical costs (55%), and total drug costs accounted for 35% of total medical costs (222,810/631,080). Table 2 also shows that the mean total drug costs of PD patients were 1.59 times higher compared to non-PD subjects (predicted values: NT$ 222,810 vs. NT$ 140,120; *p* < 0.0001). Among these costs, drug costs in outpatient care, inpatient care, and accidents were all higher in PD patients compared to non-PD subjects.

**Table 2:** Mean predicted values^1^ of medical care utilization and costs among patients with PD and non-PD^a^

Variables	Beta	SE	Standardized beta	PD patients (*N* = 50,290)		Non-PD subjects (*N* = 201,153)		*P-value*^1^
				Predicted mean	SE	Predicted mean	SE	
Frequency of Care								
Outpatient Care^b^	19.11	0.72	0.05	167.41	0.65	148.30	0.32	< .0001
Inpatient Care^c^	0.58	0.02	0.06	3.17	0.02	2.59	0.01	< .0001
Accidents (in- or outpatient)	0.89	0.03	0.05	4.62	0.03	3.73	0.01	< .0001
Hospitalization Days^d^	12.95	0.51	0.05	43.81	0.45	30.86	0.23	< .0001
Cost of Care^e^ (NT$/10^3^)								
Outpatient Care^b^	85.58	2.20	0.08	345.37	1.96	259.79	0.98	< .0001
Inpatient Care^c^	60.29	2.42	0.05	264.94	2.16	204.65	1.08	< .0001
Accidents (in- or outpatient)	4.33	0.15	0.06	20.77	0.14	16.44	0.07	< .0001
Total Medical Costs	150.21	3.60	0.08	631.08	3.21	480.88	1.60	< .0001
Drug cost (NT$/10^3^)								
Outpatient Care^b^	74.68	1.15	0.13	185.46	1.03	110.78	0.51	< .0001
Inpatient Care^c^	7.66	0.40	0.04	35.64	0.36	27.98	0.18	< .0001
Accidents (in- or outpatient)	0.36	0.02	0.03	1.72	0.02	1.36	0.01	< .0001
Total Drug Costs	82.70	1.28	0.13	222.81	1.14	140.12	0.57	< .0001

Betas represent the difference in costs between PD and non-PD subjects. Standardized betas allow to compare differences for the different costs

*SE* Standard error

^a^The average length of follow-up was 5.92 years for PD cases and 6.55 years for non-PD cases. The total follow-up person years was 297,711.19 for PD cases and 1,317,839.29 for non-PD cases

^b^Exclude accidents and emergency care/medical costs

^c^Exclude accidents care/medical cost

^d^Total hospitalization days including hospitalization due to accidents

^e^Costs were deflated by the consumer price index (CPI) with the bas year of 2016

^1^Models were adjusted for age, gender, urbanization status, insurance premium, and comorbidity

Table 3 shows differences in healthcare utilization and costs among mild (16,595, 33%), moderate (16,597, 33%), and severe PD patients (17,098, 34%). There was a trend of increasing frequency and costs of care with disease severity (*p* < 0.001 for all differences). Compared with mild and moderate PD patients, severe PD patients had a higher frequency of outpatient and inpatient visits. The cost of care increased with severity, with a total medical cost of NT$ 574,730 and NT$ 612,420 in patients with mild and moderate PD respectively compared to NT$ 780,740 for those with more severe PD. The mean cost of outpatient and inpatient care also increased with severity. Average drug costs of outpatient care for mild, moderate, and severe PD were NT$ 130,600, NT$ 160,880, and NT$ 278,150, respectively. The average drug costs of inpatient care for mild, moderate, and severe PD are NT$ 36,180, NT$ 36,790, and NT$ 38,370, respectively. Total drug costs for patients with mild and moderate PD were NT$ 168,320 and NT$ 199,340, and NT$ 318,680 for severe PD.

**Table 3:** Mean predicted values^1^ of medical care utilization and costs among patients with PD by disease PD severity^a^

Variables	Mild PD patients (*N* = 16,595)		Moderate PD patients (*N* = 16,597)				Severe PD patients (*N* = 17,098)			
	Predicted mean	SE	Standardized betas^b^	Predicted mean	SE	*P-value*^1^	Standardized betas^b^	Predicted mean	SE	*P-value*^1^
Frequency of Care										
Outpatient Care^c^	152.50	1.14	0.01	156.34	1.14	0.02	0.16	201.81	1.13	< .001
Inpatient Care^d^	3.09	0.03	0.01	3.16	0.03	0.13	0.06	3.58	0.03	< .001
Accidents (in- or outpatient)	4.28	0.05	0.01	4.44	0.05	0.03	0.08	5.52	0.05	< .001
Hospitalization Days^e^	42.06	1.03	0.02	47.22	1.03	0.0004	0.02	46.89	1.02	0.001
Cost of Care^f^ (NT$/10^3^)										
Outpatient Care^c^	295.20	3.42	0.03	320.08	3.41	< .0001	0.18	464.29	3.38	< .001
Inpatient Care^d^	260.46	4.64	0.01	272.11	4.63	0.08	0.02	291.32	4.59	< .001
Accidents (in- or outpatient)	19.07	0.26	0.02	20.23	0.26	0.002	0.08	25.13	0.26	< .001
Total Medical Costs	574.73	6.22	0.02	612.42	6.21	< .0001	0.12	780.74	6.15	< .001
Drug cost (NT$/10^3^)										
Outpatient Care^c^	130.60	1.63	0.06	160.88	1.63	< .0001	0.31	278.15	1.61	< .001
Inpatient Care^d^	36.18	0.67	0.003	36.79	0.67	0.52	0.01	38.37	0.67	0.02
Accidents (in- or outpatient)	1.54	0.04	0.01	1.67	0.04	0.01	0.06	2.16	0.04	< .001
Total Drug Costs	168.32	1.83	0.06	199.34	1.82	< .0001	0.29	318.68	1.81	< .001

Standardized betas allow to compare patients with moderate and severe PD to patients with mild PD

*SE* Standard error

^a^The average length of follow-up was 5.16 years for Mild PD cases and 5.30 years for Moderate PD cases and 7.26 years for Sever PD cases. The total follow-up person years was 85,693.73 years for Mild PD cases and 87,924.18 years for Moderate PD cases and 124,093.29 years for Sever PD cases

^b^The reference group is Mild PD cases group

^c^Exclude accidents and emergency care/medical costs

^d^Exclude accidents care/medical costs

^e^Total hospitalization days including hospitalization due to accidents

^f^Costs were deflated by the consumer price index (CPI) with the base year of 2016

^1^Models were adjusted for age, gender, urbanization status, insurance premium, and comorbidity

**Fig. 1:**
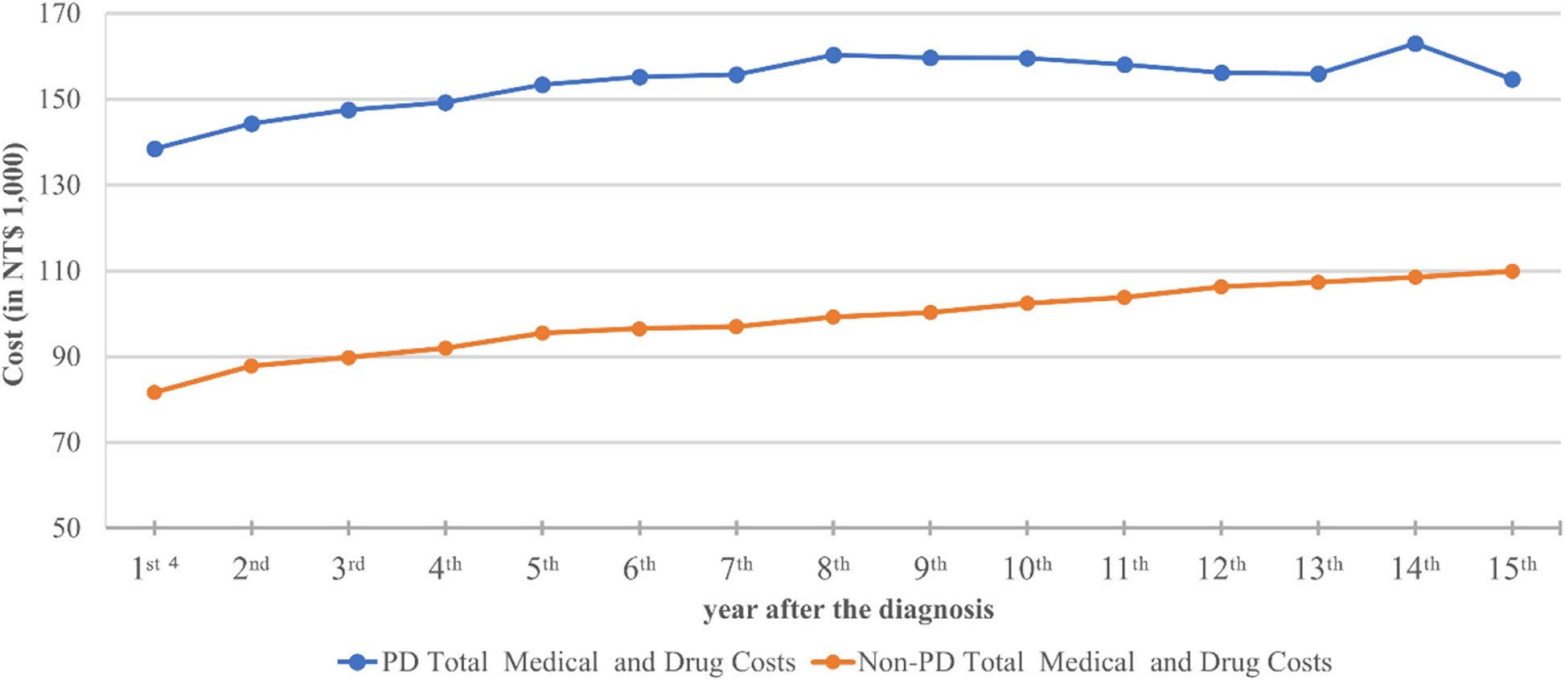
Trends in average annual medical and drug costs among patients with PD and non-PD subjects by year since diagnosis^1^ ^1^Costs were deflated by the consumer price index (CPI) with the base year of 2016

Figure 1 shows trends in costs in PD and non-PD subjects by calendar year since the index date. Total medical and drug costs for PD after diagnosis remained high, ranging from NT$138,487 per patient in the first year after diagnosis to NT$154,676 per patient in the 15th year. Moreover, expenditures were significantly higher in PD than non-PD subjects each year.

**Fig. 2:**
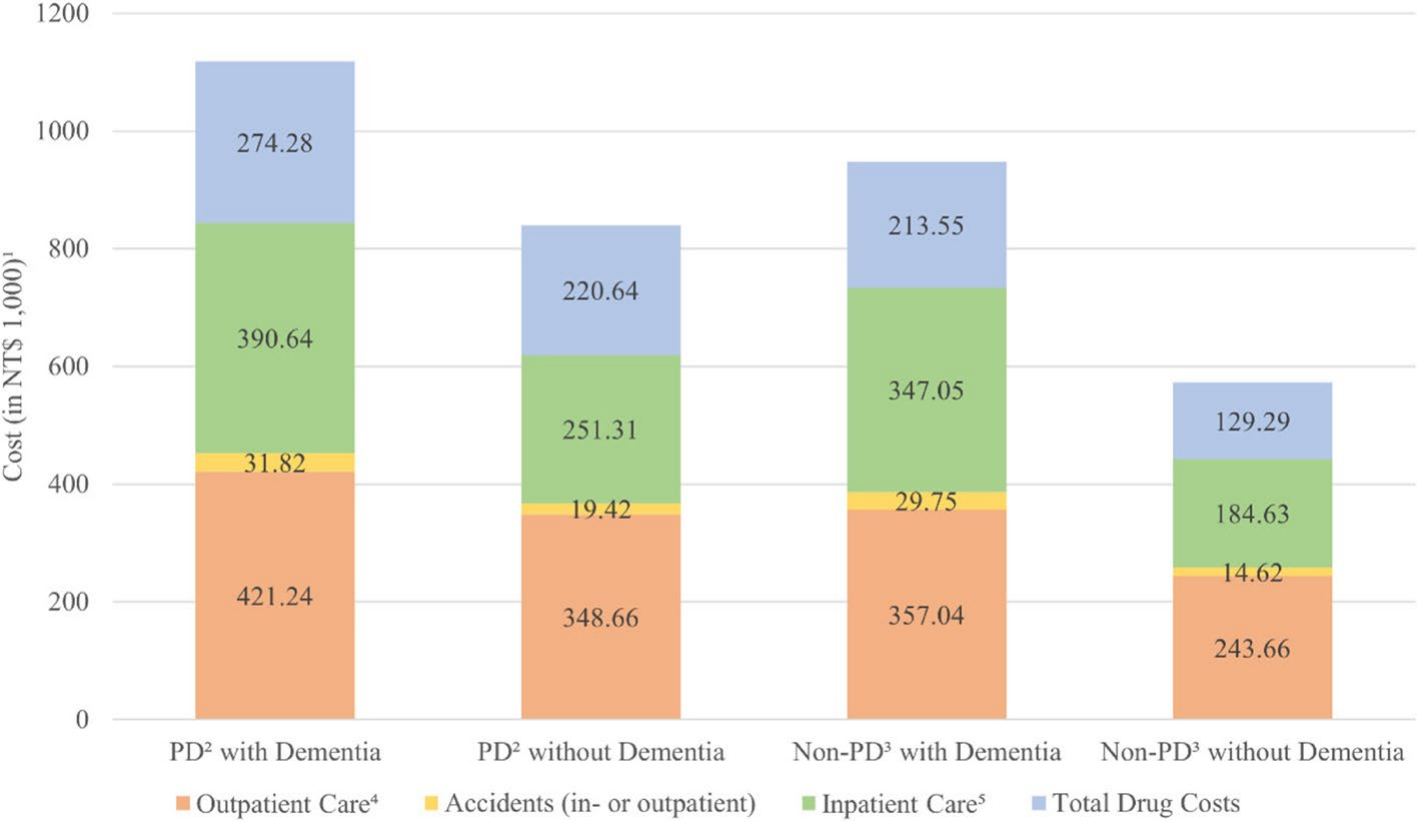
Average medical costs among PD patients and non-PD subjects according to the presence of dementia^1^ ^1^Costs were deflated by the consumer price index (CPI) with the base year of 2016. ^2^Average costs were measured among PD patients with dementia recorded at any point follow-up and among PD patients without dementia between the index date and end of 2018. ^3^Average costs were measured among Non-PD patients with dementia recorded at any point follow-up and among Non-PD patients without dementia between the index date and end of 2018. ^4^Exclude emergency and accident medical costs. ^5^Exclude accident medical cost

Of the study sample, 8,478 PD patients (incident rate = 2.85%) and 21,742 non-PD subjects (incident rate = 1.65%) developed dementia. PD patients who developed dementia had higher total medical costs than those without dementia (NT$ 843,700 vs. NT$ 619,390); costs were higher for outpatient care, accidents, and inpatient care (Fig. 2). In addition, PD patients with dementia had higher total medical costs than non-PD subjects both with and without dementia (NT$ 733,840 and NT$ 442,910). The average total drug cost of PD cases with dementia was also higher than that of patients without dementia (NT$ 274,280 vs. NT$ 220,640). Moreover, the average total drug cost of PD cases with dementia is higher than that of non-PD with and without dementia (NT$ 213,550 and NT$ 129,290).

The predicted values of medical care utilization and costs among PD patients and non-PD subjects stratified by age, gender, and duration of follow-up are shown in Appendix Table 1. In all strata, there were statistically significant differences in medical care utilization and costs between PD and non-PD subjects. Except for outpatient care, the frequency and cost of care of older PD patients were higher than those of younger PD patients. Alternatively, the total drug cost of younger PD patients was higher than that of older PD patients, mainly driven by a high drug cost in outpatient care for younger PD patients. After the 5th year of disease diagnosis, the total drug costs among younger PD patients were also higher than those with older age at diagnosis (see Appendix Figures 2). The frequency and cost of care of male PD patients were higher than that of female PD patients.

Within each stratification group (age, sex, duration of follow-up), we repeated the analyses according to disease severity of PD (see Appendix Table 2). The frequency of care, costs of care, and drug costs tended to increase with PD severity in all strata. Among 50,290 PD patients, 20,466 died and 29,824 survived. Those who died were older at baseline and more often male; they had lower insurance premium, lived more in satellite cities/tow, and had a higher CCI (Appendix Table 3). The predicted values of medical care utilization and costs among PD patients by survival status, are presented in Appendix Table 4. Compared with surviving PD patients, deceased PD patients had a higher mean predicted frequency of care, except for outpatient care. In each subcategory of costs, costs of care of deceased PD patients were higher than those of surviving PD patients. Among deceased PD patients, stratified only by age and gender, the frequency of care, costs of care, and drug costs tended to increase with PD severity (Appendix Table 5).

## Discussion

As part of a population-based cost analysis of PD, we evaluated the long-term healthcare utilization and related costs of PD patients in Taiwan over a maximum follow-up of 15 years. To our knowledge, the cost of drugs for PD patients has rarely been investigated, because most previous studies focused on total medical costs. In our study, PD cases incurred 1.31 times and 1.59 times higher total medical costs and drug costs, respectively compared with non-PD subjects. The estimated total medical costs and drug costs per PD case amounted to NT$631,080 (US$20,079) and NT$ 222,810 (US$ 7,089),^1^ which are much higher than costs incurred by non-PD subjects of NT$ 480,880 (US$ 15,300) and NT$ 140,120 (US$ 4,458) respectively. The total medical and drug cost of PD after diagnosis remained high throughout the follow-up, and increased from NT$ 138,487 (US$ 4,406) per patient in the first year following diagnosis to NT$154,676 (US$4,921) per patient in the 15th year.

Studies of the cost of PD in Asian countries other than Taiwan show that PD is a serious economic burden in those countries as well. The total mean annual health-care cost for PD in Shanghai, China, between April 2004 and March 2005 was averaged approximately to US$ 925, of which direct medical care costs represented the major component (US$ 519) [18]. However, in China the annual average cost on medical per PD patient was US$ 3,226 in 2015 [5], similar to that reported in our study. In India, the annual direct costs of managing PD were equivalent to US$ 204 [19]. In Singapore, the economic annual cost of a patient with PD was US$ 10,130 (SG$ 11,345) [3, 11], while the direct medical cost was equivalent to US$2,277 (SG$ 2,550) [6].

Over 15 years of follow-up of a cohort of PD patients, we estimated the total long-term medical costs and drug costs as US$ 20,070 (NT$ 631,080) and US$ 7,086 (NT$ 222,810) respectively. The long-term total medical and drug cost of PD was approximately six times higher than the annual cost (e.g., (631,080 + 222,810)/154,112). A similar ratio was found in a study in Singapore [11]. However, they reported a total lifetime economic burden of PD of US$ 54,011 (SG$ 60,487), which is much higher than PD-related costs in this study (US$ 35,525). Although the actual amount varies due to differences across research methods and countries, the main burden items are still consistent with the literature. In the United States, the largest part of the average cost per PD patient is for hospitalizations and nursing homes, followed by drug costs [20]. The largest average annual expenditure for PD patients in Shanghai, China, comes from drug costs [21].

In this study, the largest proportion of healthcare costs was driven by outpatient care, which differed from other countries. Taiwan’s National Health Insurance (NHI) system is a universal single-payer model that offers comprehensive benefits covering inpatient, outpatient, drug, and dental services. Importantly, the NHI system lacks a gatekeeping mechanism, allowing patients to visit any clinic or hospital directly without needing a primary care referral. This structure improves accessibility but can also lead to higher outpatient service use and potential inefficiencies compared to gatekeeping systems like the UK’s NHS. Empirical evidence from Taiwan supports that outpatient costs are often higher than inpatient costs for conditions like Alzheimer’s disease, largely due to the convenience and low out-of-pocket expenses associated with outpatient care [22]. Consequently, outpatient expenses constitute a significant portion of total medical costs.

Given the high outpatient care costs observed in this study, implementing early screening and tailored outpatient management for PD could enable timely interventions that slow disease progression and reduce the need for intensive, costly inpatient care at later stages. Research shows that early treatment can improve quality of life and lower long-term costs by addressing symptoms before they worsen [23]. Additionally, multidisciplinary teams—including neurologists, physical therapists, and occupational therapists—can provide coordinated support, reducing healthcare visits while addressing multiple health needs, potentially preventing complications that might otherwise require hospitalization [24]. Finally, educating patients and caregivers on symptom management and preventive measures can empower patients to manage their condition at home, potentially reducing emergency visits and inpatient admissions [7].

In addition, the total drug cost of younger PD patients was higher than that of older PD patients. We further analyzed the yearly average drug costs among younger and older PD patients and we found that after the 5th year of PD diagnosis, the annual average drug costs were higher in younger patients than those in older patients. This result may indicate that the higher drug cost among younger patients may not due to longer term of follow-up, rather this result may reflect the different treatment strategies between different ages of disease onset.

Younger patients tend to incur higher outpatient costs, primarily due to more frequent outpatient visits. This is likely a result of treatment strategies tailored to younger patients, who often require more intensive management, including frequent medication adjustments to maintain functionality and quality of life. For example, younger patients are often prescribed dopamine agonists early in treatment to delay levodopa-related complications, which can increase outpatient medication costs, as supported by prior research on treatment strategies in early-onset PD [12]. Additionally, while deep brain stimulation (DBS) was reimbursed starting in 2015, the small number of patients undergoing DBS (*n* = 190) is unlikely to skew the cost analysis significantly. As noted, we performed additional analyses excluding these cases, that confirmed that this subgroup did not materially alter cost trends. Lastly, we further analyzed the costs by disease severity, using the levodopa equivalent dose as a proxy for severity. Results show that costs increase substantially with disease progression, consistent with prior studies [8, 12].

In line with previous studies [10, 12, 18, 23], the group classified as having severe PD incurred greater mean costs than those classified as having mild and moderate PD. This finding held true across each subcategory of medical care we examined (outpatient care, inpatient care, accidents). The same result was also true for drug costs. Hence, as total medical and drug costs increased with disease severity, the economic burden of PD will increase in the future given the increasing prevalence of PD in aging populations. As PD progresses, costs increased markedly, placing an economic burden on the health care system, society, and the patients themselves. In Taiwan, outpatient care represents the largest component of direct costs, with prescription drugs being the second largest contributor. This is likely explained by the specificities of Taiwan’s NHI system that provides convenient and low-cost medical services. Although the costs of health care for PD patients vary by country due to different health care systems, our results revealed higher long-term health care utilization among PD patients compared to non-PD subjects.

Our findings reveal that healthcare costs rise significantly with PD severity, highlighting important implications for future resource allocation. Early diagnosis and intervention may help slow disease progression, potentially reducing the financial burdens associated with severe PD stages [12, 24]. Resource allocation should therefore prioritize specialized needs in advanced PD care, including outpatient, inpatient, and rehabilitation services, as these areas often account for the largest cost components in late-stage PD [8]. Given the anticipated rise in PD prevalence due to an aging population, it is essential for policymakers to consider long-term budget planning to effectively manage the growing economic burden of PD [7].

This study showed that the total medical costs of PD with duration of follow-up ≥ 10 years were 2.28 times higher than those with duration of follow-up 0–4 years, while the total drug costs were 3.17 times higher. In addition, the total medical costs of deceased PD patients were 1.68 times higher than that of surviving PD patients. The largest difference was for inpatient care (3.25 times higher medical costd; 4.78 times higher drug costs). The economic burden of deceased PD patients is particularly heavy on hospitalization, regardless of total medical or drug costs. Our estimates in deceased PD patients, who were on average 76.72 years old at baseline, provide estimates of the lifelong cost of PD patients. To our knowledge, the cost analysis for PD has never been performed in Taiwan, and the main purpose of this study is to utilize Taiwan’s NHI database to conduct a population-based assessment of the economic burden of PD. As the prevalence of PD is projected to grow substantially over the next few decades [7], it is important for informed decision makers in healthcare policies and plans to understand the economic burden of PD.

In this study, we considered the timing of dementia onset relative to the PD index date, as dementia significantly impacts healthcare costs. Patients who developed dementia after the PD index date incurred notably higher medical and drug costs than those without dementia, in agreement with other studies [7, 12], especially in outpatient and inpatient care. By comparing PD patients with and without dementia, our findings underscore that dementia onset imposes a substantial economic burden. Additionally, earlier dementia onset relative to PD diagnosis is associated with higher cumulative healthcare expenses, as these patients often require additional support and medical resources [24].

The strength of this study is the use of a large representative population-based dataset to track costs through long-term data. The large sample size also enabled analyses stratified by age, gender and the duration of follow-up. Our study also has limitations. First, the NHI program does not cover costs in long-term care facilities, which represent the largest component of household burden of medical insurance beneficiaries. Failure to address these costs in our study likely led to underestimate the economic burdens of PD, in particular in the oldest participants. First, this study excludes the long-term care costs, which likely leads to an underestimation of the true economic burden of PD, particularly for severe cases where long-term care needs are substantial. The lack of long-term care data in Taiwan’s NHI database restricts our ability to capture these expenses. Previous studies indicate that long-term care costs can represent a significant portion of total costs for advanced PD cases [7, 24, 25], highlighting the need for future research to include these costs for a more comprehensive assessment of PD’s economic impact. Second, this study does not account for factors such as comorbidities and functional status, which could potentially influence the classification of PD severity. While our focus is on healthcare costs associated with PD severity, future research could benefit from incorporating these additional variables. Such factors may further elucidate the drivers of healthcare utilization and outcomes, offering a more nuanced understanding of cost variations among PD patients at different severity levels. Third, the algorithm we used to determine disease severity that is based solely on medication dosage has potential limitations. As shown by Dahodwala et al. (2020) [12], contrary to expectations for disease progression, the proportion of older patients in the advanced PD group was lower, which may reflect the fact that the algorithm would not detect older patients with advanced disease who cannot tolerate higher doses of dopaminergic therapy. Forth, the nationwide health insurance in Taiwan started to reimburse the cost of deep brain stimulation (DBS) in 2015. In our analyses, we did not separate the cost of DBS from the cost of care. Given the small number of patients who underwent DBS in our study over the study period (*N* = 190, 0.4%), this is unlikely to affect considerably our estimates; we checked that excluding these patients had a small impact (data not shown). Fifth, we excluded persons younger than 35 years to avoid diagnostic errors. We considered that patients younger than 35 years who receive antiparkinsonian drugs are more likely to be treated for other conditions (e.g., dopa-responsive dystonia). Given the extremely low frequency of PD below 35 years of age, exclusion of these cases is unlikely to have a strong impact. Last, in our analysis, we used the CCI as a summary measurement of comorbidity, and we do not have detailed clinical information on comorbidities. Finally, the limitation of the NHIRD is its lack of granular clinical details, which constrains analyses that require comprehensive clinical data. Additionally, as the NHIRD is heavily dependent on diagnostic coding, the accuracy of coding practices could introduce biases if coding errors or variations exist [26].

## Conclusion

This study used a nationwide database to estimate the long-term medical utilization and expenses of PD patients in Taiwan. PD is associated with a significant economic burden, with an estimated total medical cost of NT$ 631,080 and total drug cost of NT$ 222,810, which are 1.31 and 1.59 times, respectively, higher than matched non-PD subjects. This disease not only causes a substantial cost impact on the NHI system, but also imposes a heavy economic burden on patients and their families. Moreover, costs escalated with disease severity suggesting that the burden to society is likely to grow with the increasing disease prevalence that is associated with population ageing. It is hoped that the results of this study can serve as a reference for the patient management of the government’s formulation of related policies on healthcare services for PD patients.

## Supplementary Information

Supplementary Material 1: Appendix Figure 1. Identified PD patients in the NHIRD as those with a PD code according to the International Classification of Disease (9th Revision, Clinical Modification, ICD-9-CM, code 332.0; ICD-10-CM code G20) and at least three medical claims between 2000 and 2018. According to the inclusion and exclusion criteria, we have incident PD cases (*N* = 51,140) between 2003 and 2016. To select a comparison cohort, we used risk-set sampling (4:1 ratio) to identify non-PD subjects who were matched to PD cases on age, gender, year of PD diagnosis, and city/county of residence at the index date. After further exclusion criteria, the final sample included 251,433 participants (50,290 PD cases, 201,153 non-PD subjects). Appendix Figure 2. Shows trends in average annual medical and drug costs since diagnosis among PD patients < 72 years and ≧ 72 years and their control group. There were significant differences in medical care utilization and costs between PD and non-PD subjects, whether younger or older. However, after the 5th year of disease diagnosis, the expenditures among younger PD patients were higher than those with older age at diagnosis. Appendix Table 1. Shows the predicted values of medical care utilization and costs among patients with PD and non-PD Stratified by age, gender, and duration of follow-up. Except for outpatient care, the frequency and cost of care of older PD patients were higher than those of younger PD patients. Alternatively, the total drug cost of younger PD patients was higher than that of older PD patients. After the 5th year of disease diagnosis, the total drug costs among younger PD patients were also higher than those with older age at diagnosis. The frequency and cost of care of male PD patients were higher than that of female PD patients. Appendix Table 2. Shows the predicted values of medical care utilization and costs among patients with different severity of PD stratified by age, gender, and duration of follow-up. The frequency of care, costs of care, and drug costs tended to increase with PD severity in all strata. Appendix Table 3. Shows the sociodemographic characteristics of deceased and survived PD participants. Among 50,290 PD patients, 20,466 died and 29,824 survived. Those who died were older at baseline and more often male; they had lower insurance premium, lived more in satellite cities/tow, and had a higher CCI. Appendix Table 4. Shows the predicted values of medical care utilization and costs among patients with PD stratified by survival status. Compared with surviving PD patients, deceased PD patients had a higher mean predicted frequency of care, except for outpatient care. In each subcategory of costs, costs of care of deceased PD patients were higher than those of surviving PD patients. Appendix Table 5. Shows predicted values of medical care utilization and costs among deceased cases with different severity of PD stratified by age, gender, and duration of follow-up. Among deceased PD patients, stratified only by age and gender, the frequency of care, costs of care, and drug costs tended to increase with PD severity.

## Data Availability

All data are stored at the Health and Welfare Data Science Center.

## Abbreviations

PD: Parkinson’s Disease
NHI: National Health Insurance
NHIRD: National Health Insurance Research Database
CPI: Consumer Price Index
CCI: Charlson Comorbidity Index
DBS: Deep Brain Stimulation
ATC: Anatomical Therapeutic Chemical (classification system)
GLM: Generalized Linear Model
UPDRS: Unified Parkinson’s Disease Rating Scale
SD: Standard Deviation
SE: Standard Error
ICD: International Classification of Diseases
US$: United States Dollar
NT$: New Taiwan Dollar
IRB: Institutional Review Board
HREC: Human Research Ethics Committee

## Footnotes

1 All costs were converted to US dollars at the average exchange rate (1USD = 31.43NTD) from 2005 to 2016.
