# Supplementary material for: Healthcare utilization and costs for patients with Parkinson’s disease in Taiwan
Source: BMC Neurol. 2025 Jan 3;25:3. doi: 10.1186/s12883-024-03988-3 (PMC11697947; doi:10.1186/s12883-024-03988-3)
Supplement: Supplementary file 1 — Supplementary Material 1: Appendix Figure 1. Identified PD patients in the NHIRD as those with a PD code according to the International Classification of Disease (9th Revision, Clinical Modification, ICD-9-CM, code 332.0; ICD-10-CM code G20) and at least three medical claims between 2000 and 2018. According to the inclusion and exclusion criteria, we have incident PD cases (N = 51,140) between 2003 and 2016. To select a comparison cohort, we used risk-set sampling (4:1 ratio) to identify non-PD subjects who were matched to PD cases on age, gender, year of PD diagnosis, and city/county of residence at the index date. After further exclusion criteria, the final sample included 251,433 participants (50,290 PD cases, 201,153 non-PD subjects). Appendix Figure 2. Shows trends in average annual medical and drug costs since diagnosis among PD patients < 72 years and ≧ 72 years and their control group. There were significant differences in medical care utilization and costs between PD and non-PD subjects, whether younger or older. However, after the 5th year of disease diagnosis, the expenditures among younger PD patients were higher than those with older age at diagnosis. Appendix Table 1. Shows the predicted values of medical care utilization and costs among patients with PD and non-PD Stratified by age, gender, and duration of follow-up. Except for outpatient care, the frequency and cost of care of older PD patients were higher than those of younger PD patients. Alternatively, the total drug cost of younger PD patients was higher than that of older PD patients. After the 5th year of disease diagnosis, the total drug costs among younger PD patients were also higher than those with older age at diagnosis. The frequency and cost of care of male PD patients were higher than that of female PD patients. Appendix Table 2. Shows the predicted values of medical care utilization and costs among patients with different severity of PD stratified by age, gender, and duration of follow [file 12883_2024_3988_MOESM1_ESM.docx]

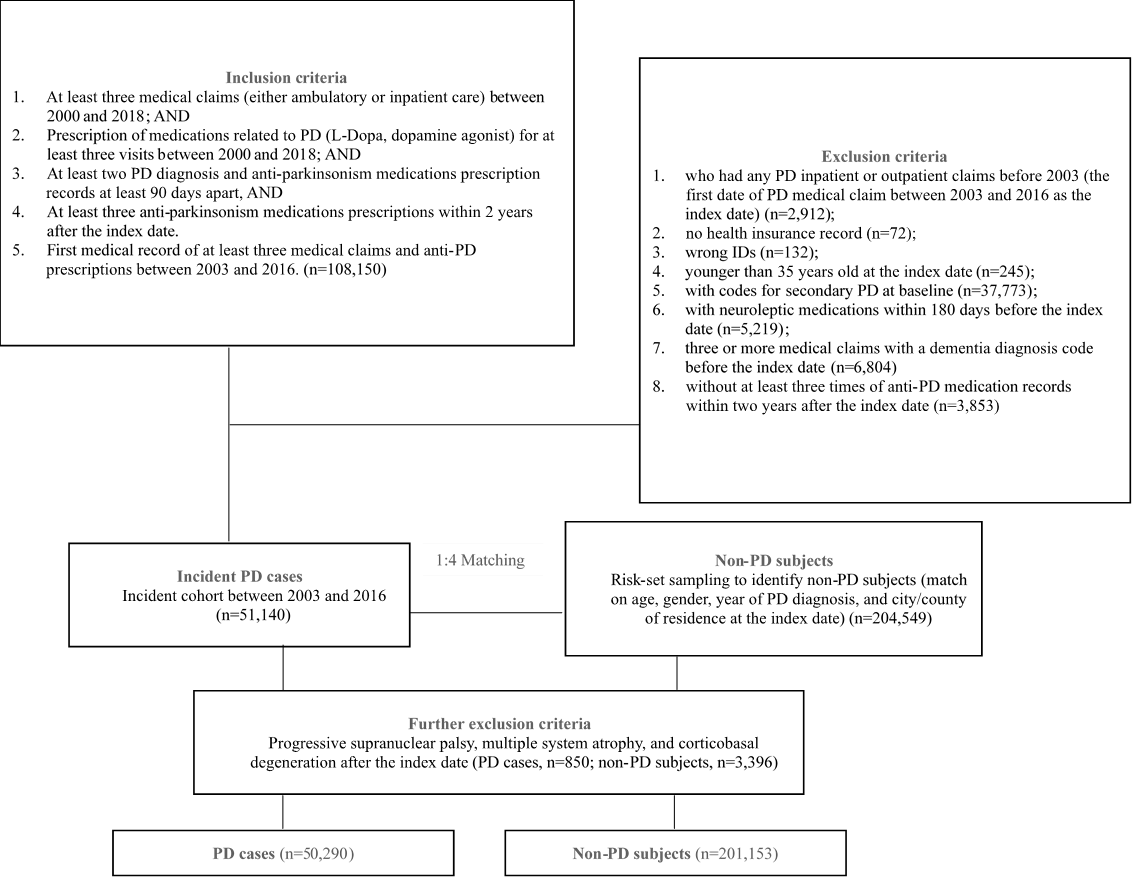


**Appendix Figure 1 Flow-chart for the identification of Parkinson's disease (PD) incident cases and non-PD subjects**


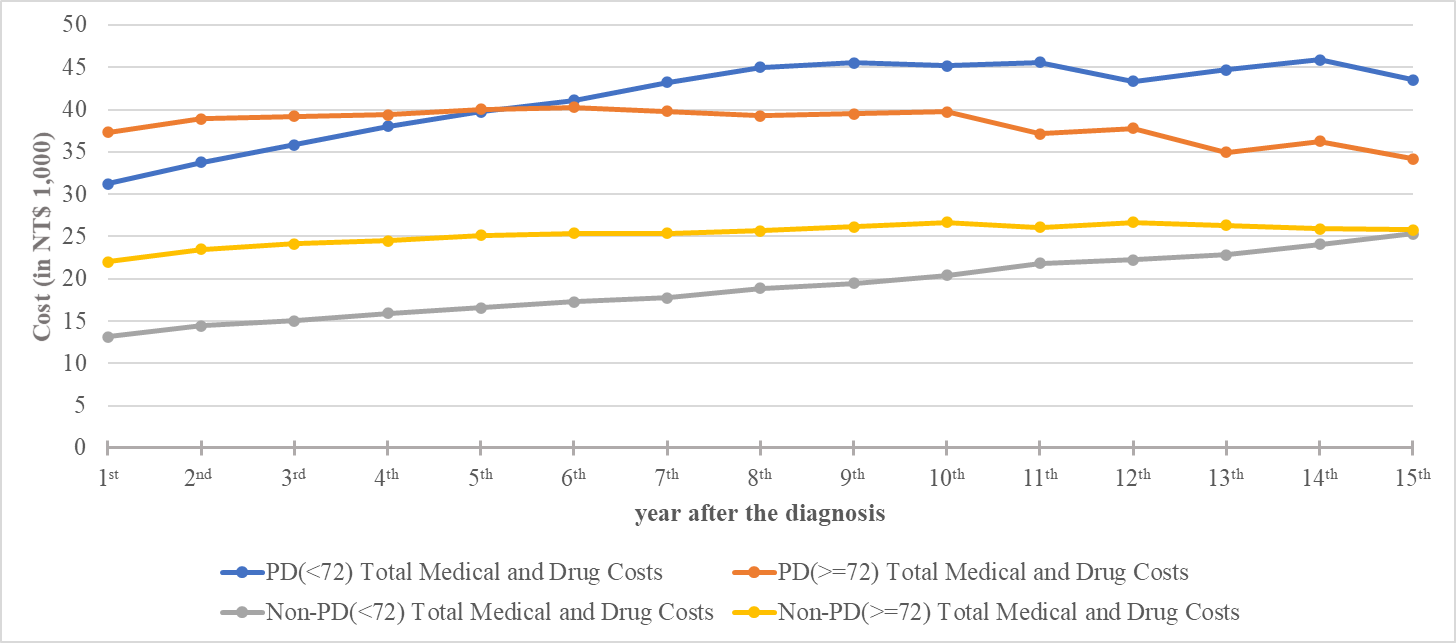


**Appendix Figure** **2 Trends in Average Annual Medical and Drug Costs Since Diagnosis Among PD Patients < 72 Years and ≧ 72 Years and their Control Group**

**Appendix Table 1** Mean Predicted Values^1^ of Medical Care Utilization and Costs Among Patients With PD and Non-PD Stratified by Age, Gender, and Duration of Follow-up

|  |  |  |  | PD cases  (N=50,290) | | Non-PD (N=201,153) | |  |
| --- | --- | --- | --- | --- | --- | --- | --- | --- |
|  | Beta | SE | Standardized betas | predicted mean | SE | predicted mean | SE | *p-value*^1^ |
| Age (<72) |  |  |  |  |  |  |  |  |
| Frequency of Care |  |  |  |  |  |  |  |  |
| Outpatient Care^2^ | 27.31 | 1.15 | 0.07 | 172.38 | 1.03 | 145.06 | 0.51 | <.001 |
| Inpatient Care^3^ | 0.63 | 0.03 | 0.07 | 2.51 | 0.02 | 1.88 | 0.01 | <.001 |
| Accidents (in- or outpatient) | 1.04 | 0.04 | 0.07 | 3.74 | 0.04 | 2.70 | 0.02 | <.001 |
| Hospitalization Days^4^ | 15.00 | 0.75 | 0.06 | 34.02 | 0.67 | 19.02 | 0.33 | <.001 |
| Cost of Care^5^ (NT$/10^3^) |  |  |  |  |  |  |  |  |
| Outpatient Care^2^ | 138.55 | 3.90 | 0.11 | 395.18 | 3.48 | 256.63 | 1.73 | <.001 |
| Inpatient Care^3^ | 65.59 | 3.23 | 0.06 | 206.53 | 2.88 | 140.93 | 1.43 | <.001 |
| Accidents (in- or outpatient) | 4.70 | 0.22 | 0.07 | 15.70 | 0.19 | 11.00 | 0.10 | <.001 |
| Total Medical Costs | 208.84 | 5.70 | 0.11 | 617.40 | 5.09 | 408.57 | 2.53 | <.001 |
| Drug cost (NT$/10^3^) |  |  |  |  |  |  |  |  |
| Outpatient Care^2^ | 116.05 | 2.09 | 0.17 | 221.03 | 1.86 | 104.98 | 0.93 | <.001 |
| Inpatient Care^2^ | 6.20 | 0.63 | 0.03 | 25.29 | 0.56 | 19.10 | 0.28 | <.001 |
| Accidents (in- or outpatient) | 0.31 | 0.04 | 0.03 | 1.19 | 0.03 | 0.88 | 0.02 | <.001 |
| Total Drug Costs | 122.56 | 2.31 | 0.16 | 247.51 | 2.06 | 124.96 | 1.02 | <.001 |
| Age (≧72) |  |  |  |  |  |  |  |  |
| Frequency of Care |  |  |  |  |  |  |  |  |
| Outpatient Care^2^ | 12.85 | 0.92 | 0.04 | 163.48 | 0.82 | 150.63 | 0.41 | <.001 |
| Inpatient Care^3^ | 0.52 | 0.03 | 0.05 | 3.59 | 0.02 | 3.07 | 0.01 | <.001 |
| Accidents (in- or outpatient) | 0.76 | 0.05 | 0.04 | 5.19 | 0.04 | 4.42 | 0.02 | <.001 |
| Hospitalization Days^4^ | 11.37 | 0.69 | 0.04 | 50.22 | 0.61 | 38.86 | 0.31 | <.001 |
| Cost of Care^5^ (NT$/10^3^) |  |  |  |  |  |  |  |  |
| Outpatient Care^2^ | 47.43 | 2.56 | 0.05 | 309.86 | 2.29 | 262.43 | 1.14 | <.001 |
| Inpatient Care^3^ | 55.02 | 3.42 | 0.04 | 302.80 | 3.05 | 247.78 | 1.52 | <.001 |
| Accidents (in- or outpatient) | 3.93 | 0.21 | 0.05 | 24.05 | 0.19 | 20.12 | 0.09 | <.001 |
| Total Medical Costs | 106.37 | 4.61 | 0.06 | 636.71 | 4.12 | 530.34 | 2.06 | <.001 |
| Drug cost (NT$/10^3^) |  |  |  |  |  |  |  |  |
| Outpatient Care^2^ | 46.27 | 1.31 | 0.09 | 161.07 | 1.17 | 114.81 | 0.58 | <.001 |
| Inpatient Care^3^ | 8.37 | 0.52 | 0.04 | 42.37 | 0.46 | 34.00 | 0.23 | <.001 |
| Accidents (in- or outpatient) | 0.38 | 0.03 | 0.03 | 2.06 | 0.03 | 1.68 | 0.01 | <.001 |
| Total Drug Costs | 55.01 | 1.46 | 0.10 | 205.50 | 1.31 | 150.48 | 0.65 | <.001 |
| Gender (Female) |  |  |  |  |  |  |  |  |
| Frequency of Care |  |  |  |  |  |  |  |  |
| Outpatient Care^2^ | 23.56 | 1.04 | 0.06 | 178.24 | 0.93 | 154.68 | 0.46 | <.001 |
| Inpatient Care^3^ | 0.60 | 0.03 | 0.07 | 2.93 | 0.02 | 2.33 | 0.01 | <.001 |
| Accidents (in- or outpatient) | 0.93 | 0.04 | 0.06 | 4.42 | 0.04 | 3.50 | 0.02 | <.001 |
| Hospitalization Days^4^ | 12.18 | 0.69 | 0.05 | 38.69 | 0.62 | 26.51 | 0.31 | <.001 |
| Cost of Care^5^ (NT$/10^3^) |  |  |  |  |  |  |  |  |
| Outpatient Care^2^ | 94.21 | 3.18 | 0.08 | 357.26 | 2.84 | 263.05 | 1.42 | <.001 |
| Inpatient Care^3^ | 59.00 | 3.24 | 0.05 | 235.10 | 2.89 | 176.10 | 1.44 | <.001 |
| Accidents (in- or outpatient) | 4.14 | 0.22 | 0.05 | 19.36 | 0.19 | 15.22 | 0.10 | <.001 |
| Total Medical Costs | 157.36 | 5.03 | 0.09 | 611.72 | 4.49 | 454.37 | 2.24 | <.001 |
| Drug cost (NT$/10^3^) |  |  |  |  |  |  |  |  |
| Outpatient Care^2^ | 79.41 | 1.48 | 0.15 | 188.12 | 1.32 | 108.71 | 0.66 | <.001 |
| Inpatient Care^3^ | 6.70 | 0.50 | 0.04 | 28.64 | 0.44 | 21.94 | 0.22 | <.001 |
| Accidents (in- or outpatient) | 0.31 | 0.03 | 0.03 | 1.44 | 0.02 | 1.13 | 0.01 | <.001 |
| Total Drug Costs | 86.42 | 1.63 | 0.15 | 218.20 | 1.46 | 131.78 | 0.73 | <.001 |
| Gender (Male) |  |  |  |  |  |  |  |  |
| Frequency of Care |  |  |  |  |  |  |  |  |
| Outpatient Care^2^ | 14.83 | 1.00 | 0.04 | 157.12 | 0.89 | 142.29 | 0.45 | <.001 |
| Inpatient Care^3^ | 0.57 | 0.03 | 0.06 | 3.39 | 0.02 | 2.83 | 0.01 | <.001 |
| Accidents (in- or outpatient) | 0.86 | 0.05 | 0.05 | 4.80 | 0.04 | 3.95 | 0.02 | <.001 |
| Hospitalization Days^4^ | 13.67 | 0.74 | 0.05 | 48.65 | 0.67 | 34.98 | 0.33 | <.001 |
| Cost of Care^5^ (NT$/10^3^) |  |  |  |  |  |  |  |  |
| Outpatient Care^2^ | 77.55 | 3.04 | 0.07 | 334.24 | 2.72 | 256.69 | 1.35 | <.001 |
| Inpatient Care^3^ | 61.51 | 3.58 | 0.05 | 293.14 | 3.20 | 231.63 | 1.59 | <.001 |
| Accidents (in- or outpatient) | 4.52 | 0.21 | 0.06 | 22.11 | 0.19 | 17.59 | 0.09 | <.001 |
| Total Medical Costs | 143.58 | 5.13 | 0.08 | 649.49 | 4.59 | 505.91 | 2.29 | <.001 |
| Drug cost (NT$/10^3^) |  |  |  |  |  |  |  |  |
| Outpatient Care^2^ | 70.09 | 1.75 | 0.11 | 182.85 | 1.56 | 112.76 | 0.78 | <.001 |
| Inpatient Care^3^ | 8.57 | 0.62 | 0.04 | 42.26 | 0.56 | 33.69 | 0.28 | <.001 |
| Accidents (in- or outpatient) | 0.41 | 0.04 | 0.03 | 1.98 | 0.03 | 1.57 | 0.02 | <.001 |
| Total Drug Costs | 79.07 | 1.95 | 0.11 | 227.09 | 1.74 | 148.02 | 0.87 | <.001 |
| Duration of follow up (<5 years) |  |  |  |  |  |  |  |  |
| Frequency of Care |  |  |  |  |  |  |  |  |
| Outpatient Care^2^ | 18.03 | 0.48 | 0.11 | 91.12 | 0.42 | 73.08 | 0.22 | <.001 |
| Inpatient Care^3^ | 0.49 | 0.02 | 0.07 | 2.45 | 0.02 | 1.95 | 0.01 | <.001 |
| Accidents (in- or outpatient) | 0.79 | 0.03 | 0.08 | 3.19 | 0.03 | 2.39 | 0.01 | <.001 |
| Hospitalization Days^4^ | 9.06 | 0.51 | 0.05 | 34.74 | 0.45 | 25.69 | 0.24 | <.001 |
| Cost of Care^5^ (NT$/10^3^) |  |  |  |  |  |  |  |  |
| Outpatient Care^2^ | 45.99 | 2.02 | 0.07 | 197.01 | 1.78 | 151.03 | 0.95 | <.001 |
| Inpatient Care^3^ | 47.23 | 2.91 | 0.05 | 224.18 | 2.57 | 176.96 | 1.37 | <.001 |
| Accidents (in- or outpatient) | 3.66 | 0.17 | 0.06 | 15.51 | 0.15 | 11.84 | 0.08 | <.001 |
| Total Medical Costs | 96.88 | 3.82 | 0.07 | 436.70 | 3.37 | 339.83 | 1.79 | <.001 |
| Drug cost (NT$/10^3^) |  |  |  |  |  |  |  |  |
| Outpatient Care^2^ | 33.55 | 1.14 | 0.09 | 95.67 | 1.00 | 62.13 | 0.53 | <.001 |
| Inpatient Care^3^ | 6.39 | 0.56 | 0.03 | 33.81 | 0.49 | 27.42 | 0.26 | <.001 |
| Accidents (in- or outpatient) | 0.27 | 0.04 | 0.02 | 1.35 | 0.03 | 1.08 | 0.02 | <.001 |
| Total Drug Costs | 40.21 | 1.32 | 0.09 | 130.84 | 1.16 | 90.63 | 0.62 | <.001 |
| Duration of follow up (5-10 years) |  |  |  |  |  |  |  |  |
| Frequency of Care |  |  |  |  |  |  |  |  |
| Outpatient Care^2^ | 33.20 | 0.99 | 0.10 | 198.69 | 0.88 | 165.49 | 0.44 | <.001 |
| Inpatient Care^3^ | 0.79 | 0.03 | 0.08 | 3.66 | 0.03 | 2.86 | 0.01 | <.001 |
| Accidents (in- or outpatient) | 1.24 | 0.05 | 0.07 | 5.41 | 0.05 | 4.17 | 0.02 | <.001 |
| Hospitalization Days^4^ | 16.34 | 0.95 | 0.06 | 50.91 | 0.85 | 34.57 | 0.42 | <.001 |
| Cost of Care^5^ (NT$/10^3^) |  |  |  |  |  |  |  |  |
| Outpatient Care^2^ | 118.43 | 3.65 | 0.10 | 411.66 | 3.26 | 293.24 | 1.62 | <.001 |
| Inpatient Care^3^ | 76.08 | 4.38 | 0.06 | 298.58 | 3.91 | 222.50 | 1.95 | <.001 |
| Accidents (in- or outpatient) | 5.58 | 0.26 | 0.07 | 23.95 | 0.24 | 18.38 | 0.12 | <.001 |
| Total Medical Costs | 200.09 | 6.20 | 0.10 | 734.20 | 5.54 | 534.11 | 2.75 | <.001 |
| Drug cost (NT$/10^3^) |  |  |  |  |  |  |  |  |
| Outpatient Care^2^ | 98.56 | 1.81 | 0.17 | 222.93 | 1.62 | 124.37 | 0.80 | <.001 |
| Inpatient Care^3^ | 8.83 | 0.70 | 0.04 | 38.28 | 0.63 | 29.45 | 0.31 | <.001 |
| Accidents (in- or outpatient) | 0.46 | 0.04 | 0.04 | 1.96 | 0.03 | 1.50 | 0.02 | <.001 |
| Total Drug Costs | 107.85 | 2.02 | 0.17 | 263.17 | 1.81 | 155.32 | 0.90 | <.001 |
| Duration of follow up (>10 years) |  |  |  |  |  |  |  |  |
| Frequency of Care |  |  |  |  |  |  |  |  |
| Outpatient Care^2^ | 52.20 | 2.39 | 0.10 | 326.64 | 2.20 | 274.44 | 0.93 | <.001 |
| Inpatient Care^3^ | 0.96 | 0.05 | 0.08 | 4.34 | 0.05 | 3.38 | 0.02 | <.001 |
| Accidents (in- or outpatient) | 1.59 | 0.12 | 0.06 | 7.28 | 0.11 | 5.68 | 0.05 | <.001 |
| Hospitalization Days^4^ | 22.24 | 1.64 | 0.06 | 56.75 | 1.50 | 34.50 | 0.64 | <.001 |
| Cost of Care^5^ (NT$/10^3^) |  |  |  |  |  |  |  |  |
| Outpatient Care^2^ | 218.91 | 7.48 | 0.13 | 645.31 | 6.87 | 426.40 | 2.91 | <.001 |
| Inpatient Care^3^ | 93.68 | 6.87 | 0.06 | 321.23 | 6.31 | 227.55 | 2.67 | <.001 |
| Accidents (in- or outpatient) | 7.89 | 0.47 | 0.08 | 30.21 | 0.43 | 22.32 | 0.18 | <.001 |
| Total Medical Costs | 320.47 | 11.18 | 0.13 | 996.75 | 10.27 | 676.28 | 4.35 | <.001 |
| Drug cost (NT$/10^3^) |  |  |  |  |  |  |  |  |
| Outpatient Care^2^ | 188.13 | 4.06 | 0.20 | 375.93 | 3.73 | 187.80 | 1.58 | <.001 |
| Inpatient Care^3^ | 10.82 | 0.99 | 0.05 | 36.88 | 0.91 | 26.06 | 0.39 | <.001 |
| Accidents (in- or outpatient) | 0.71 | 0.05 | 0.06 | 2.37 | 0.05 | 1.66 | 0.02 | <.001 |
| Total Drug Costs | 199.66 | 4.42 | 0.20 | 415.18 | 4.06 | 215.51 | 1.72 | <.001 |

SE, standard error. Betas represent the difference in costs between PD and non-PD subjects. Standardized betas allow to compare differences for the different costs.^1^ Models were adjusted for age, gender, urbanization status, insurance premium, and comorbidity. ^3^ Exclude accidents and emergency care/medical costs. ^4^ Exclude accidents care/medical cost. ^5^ Total hospitalization days including hospitalization due to accidents. ^6^ Costs were deflated by the consumer price index (CPI) with the base year of 2016.

**Appendix Table 2** Mean Predicted Values1 of Medical Care Utilization and Costs Among Patients With Different Severity of PD Stratified by Age, Gender, and Duration of Follow-up1

|  | Mild PD cases (N=16,595) | |  | Moderate PD cases  (N=16,597) | | | |  | Severe PD cases  (N=17,098) | | | |
| --- | --- | --- | --- | --- | --- | --- | --- | --- | --- | --- | --- | --- |
|  | predicted mean | SE |  | standardized betas^2^ | predicted mean | SE | *p-value*^1^ |  | standardized betas^2^ | predicted mean | SE | *p-value*^1^ |
| Age (<72) |  |  |  |  |  |  |  |  |  |  |  |  |
| Frequency of Care |  |  |  |  |  |  |  |  |  |  |  |  |
| Outpatient Care^3^ | 162.99 | 1.96 |  | −0.01 | 158.10 | 1.94 | 0.08 |  | 0.13 | 205.66 | 1.71 | <.0001 |
| Inpatient Care^4^ | 2.51 | 0.05 |  | −0.01 | 2.47 | 0.05 | 0.51 |  | 0.05 | 2.96 | 0.04 | <.0001 |
| Accidents (in- or outpatient) | 3.62 | 0.09 |  | −0.01 | 3.46 | 0.09 | 0.18 |  | 0.07 | 4.55 | 0.08 | <.0001 |
| Hospitalization Days^5^ | 32.36 | 1.82 |  | 0.02 | 37.05 | 1.80 | 0.07 |  | 0.02 | 37.81 | 1.58 | 0.02 |
| Cost of Care^6^ (NT$/10^3^) |  |  |  |  |  |  |  |  |  |  |  |  |
| Outpatient Care^3^ | 337.77 | 6.74 |  | 0.01 | 352.29 | 6.67 | 0.13 |  | 0.18 | 540.18 | 5.87 | <.0001 |
| Inpatient Care^4^ | 200.79 | 7.20 |  | 0.01 | 207.72 | 7.12 | 0.49 |  | 0.04 | 244.35 | 6.28 | <.0001 |
| Accidents (in- or outpatient) | 15.08 | 0.42 |  | 0.002 | 15.24 | 0.42 | 0.79 |  | 0.06 | 19.26 | 0.37 | <.0001 |
| Total Medical Costs | 553.64 | 10.84 |  | 0.01 | 575.25 | 10.73 | 0.16 |  | 0.14 | 803.80 | 9.45 | <.0001 |
| Drug cost (NT$/10^3^) |  |  |  |  |  |  |  |  |  |  |  |  |
| Outpatient Care^3^ | 143.05 | 3.19 |  | 0.06 | 178.59 | 3.15 | <.0001 |  | 0.36 | 335.24 | 2.78 | <.0001 |
| Inpatient Care^4^ | 26.53 | 1.13 |  | −0.004 | 25.68 | 1.12 | 0.60 |  | 0.02 | 29.36 | 0.99 | 0.06 |
| Accidents (in- or outpatient) | 1.13 | 0.05 |  | 0.003 | 1.16 | 0.05 | 0.68 |  | 0.05 | 1.51 | 0.04 | <.0001 |
| Total Drug Costs | 170.71 | 3.50 |  | 0.06 | 205.44 | 3.46 | <.0001 |  | 0.33 | 366.11 | 3.05 | <.0001 |
| Age (≧72) |  |  |  |  |  |  |  |  |  |  |  |  |
| Frequency of Care |  |  |  |  |  |  |  |  |  |  |  |  |
| Outpatient Care^3^ | 145.70 | 1.38 |  | 0.03 | 154.34 | 1.38 | <.0001 |  | 0.17 | 200.43 | 1.48 | <.0001 |
| Inpatient Care^4^ | 3.49 | 0.04 |  | 0.01 | 3.62 | 0.04 | 0.03 |  | 0.05 | 3.97 | 0.04 | <.0001 |
| Accidents (in- or outpatient) | 4.77 | 0.07 |  | 0.02 | 5.10 | 0.07 | 0.001 |  | 0.09 | 6.17 | 0.07 | <.0001 |
| Hospitalization Days^5^ | 48.80 | 1.23 |  | 0.02 | 54.07 | 1.24 | 0.003 |  | 0.01 | 52.54 | 1.33 | 0.04 |
| Cost of Care^6^ (NT$/10^3^) |  |  |  |  |  |  |  |  |  |  |  |  |
| Outpatient Care^3^ | 262.82 | 3.61 |  | 0.04 | 293.91 | 3.62 | <.0001 |  | 0.18 | 415.10 | 3.89 | <.0001 |
| Inpatient Care^4^ | 301.38 | 6.04 |  | 0.01 | 314.91 | 6.06 | 0.11 |  | 0.01 | 319.36 | 6.50 | 0.04 |
| Accidents (in- or outpatient) | 21.99 | 0.34 |  | 0.02 | 23.62 | 0.34 | 0.001 |  | 0.09 | 29.04 | 0.36 | <.0001 |
| Total Medical Costs | 586.19 | 7.47 |  | 0.03 | 632.44 | 7.49 | <.0001 |  | 0.10 | 763.51 | 8.04 | <.0001 |
| Drug cost (NT$/10^3^) |  |  |  |  |  |  |  |  |  |  |  |  |
| Outpatient Care^3^ | 118.78 | 1.77 |  | 0.07 | 146.45 | 1.77 | <.0001 |  | 0.29 | 238.05 | 1.90 | <.0001 |
| Inpatient Care^4^ | 42.93 | 0.83 |  | 0.01 | 44.19 | 0.84 | 0.29 |  | 0.01 | 43.93 | 0.90 | 0.41 |
| Accidents (in- or outpatient) | 1.85 | 0.05 |  | 0.02 | 2.01 | 0.05 | 0.02 |  | 0.07 | 2.59 | 0.05 | <.0001 |
| Total Drug Costs | 163.56 | 2.01 |  | 0.06 | 192.65 | 2.02 | <.0001 |  | 0.26 | 284.57 | 2.17 | <.0001 |
| Gender (Female) |  |  |  |  |  |  |  |  |  |  |  |  |
| Frequency of Care |  |  |  |  |  |  |  |  |  |  |  |  |
| Outpatient Care^3^ | 166.16 | 1.65 |  | 0.01 | 167.81 | 1.67 | 0.48 |  | 0.14 | 209.78 | 1.67 | <.0001 |
| Inpatient Care^4^ | 2.87 | 0.04 |  | 0.0005 | 2.88 | 0.04 | 0.95 |  | 0.05 | 3.32 | 0.04 | <.0001 |
| Accidents (in- or outpatient) | 4.11 | 0.07 |  | 0.01 | 4.28 | 0.07 | 0.10 |  | 0.08 | 5.26 | 0.07 | <.0001 |
| Hospitalization Days^5^ | 38.57 | 1.39 |  | 0.01 | 39.92 | 1.41 | 0.49 |  | 0.01 | 41.61 | 1.41 | 0.13 |
| Cost of Care^6^ (NT$/10^3^) |  |  |  |  |  |  |  |  |  |  |  |  |
| Outpatient Care^3^ | 315.12 | 5.04 |  | 0.02 | 334.59 | 5.11 | 0.01 |  | 0.15 | 467.57 | 5.10 | <.0001 |
| Inpatient Care^4^ | 235.49 | 6.32 |  | −0.001 | 234.10 | 6.41 | 0.88 |  | 0.02 | 260.43 | 6.40 | 0.01 |
| Accidents (in- or outpatient) | 18.11 | 0.37 |  | 0.01 | 19.07 | 0.38 | 0.07 |  | 0.07 | 23.00 | 0.38 | <.0001 |
| Total Medical Costs | 568.72 | 8.78 |  | 0.01 | 587.76 | 8.90 | 0.13 |  | 0.11 | 751.00 | 8.88 | <.0001 |
| Drug cost (NT$/10^3^) |  |  |  |  |  |  |  |  |  |  |  |  |
| Outpatient Care^3^ | 138.17 | 2.31 |  | 0.06 | 165.53 | 2.34 | <.0001 |  | 0.29 | 275.37 | 2.34 | <.0001 |
| Inpatient Care^4^ | 29.64 | 0.83 |  | −0.004 | 29.03 | 0.85 | 0.61 |  | 0.01 | 30.76 | 0.84 | 0.35 |
| Accidents (in- or outpatient) | 1.33 | 0.04 |  | 0.01 | 1.43 | 0.05 | 0.11 |  | 0.05 | 1.75 | 0.05 | <.0001 |
| Total Drug Costs | 169.15 | 2.54 |  | 0.05 | 196.00 | 2.58 | <.0001 |  | 0.27 | 307.87 | 2.57 | <.0001 |
| Gender (Male) |  |  |  |  |  |  |  |  |  |  |  |  |
| Frequency of Care |  |  |  |  |  |  |  |  |  |  |  |  |
| Outpatient Care^3^ | 139.57 | 1.57 |  | 0.02 | 145.43 | 1.54 | 0.01 |  | 0.18 | 194.11 | 1.52 | <.0001 |
| Inpatient Care^4^ | 3.29 | 0.05 |  | 0.01 | 3.41 | 0.05 | 0.06 |  | 0.06 | 3.82 | 0.05 | <.0001 |
| Accidents (in- or outpatient) | 4.45 | 0.08 |  | 0.01 | 4.59 | 0.08 | 0.19 |  | 0.09 | 5.77 | 0.08 | <.0001 |
| Hospitalization Days^5^ | 45.33 | 1.52 |  | 0.03 | 54.07 | 1.49 | <.0001 |  | 0.02 | 51.86 | 1.47 | 0.002 |
| Cost of Care^6^ (NT$/10^3^) |  |  |  |  |  |  |  |  |  |  |  |  |
| Outpatient Care^3^ | 276.32 | 4.63 |  | 0.03 | 306.17 | 4.55 | <.0001 |  | 0.20 | 460.73 | 4.47 | <.0001 |
| Inpatient Care^4^ | 283.94 | 6.78 |  | 0.02 | 307.71 | 6.66 | 0.01 |  | 0.03 | 320.62 | 6.55 | 0.0001 |
| Accidents (in- or outpatient) | 19.99 | 0.38 |  | 0.02 | 21.29 | 0.37 | 0.01 |  | 0.10 | 27.11 | 0.36 | <.0001 |
| Total Medical Costs | 580.24 | 8.81 |  | 0.03 | 635.17 | 8.66 | <.0001 |  | 0.13 | 808.47 | 8.52 | <.0001 |
| Drug cost (NT$/10^3^) |  |  |  |  |  |  |  |  |  |  |  |  |
| Outpatient Care^3^ | 123.28 | 2.31 |  | 0.07 | 156.49 | 2.27 | <.0001 |  | 0.33 | 280.50 | 2.23 | <.0001 |
| Inpatient Care^4^ | 42.37 | 1.05 |  | 0.01 | 44.04 | 1.03 | 0.26 |  | 0.02 | 45.59 | 1.02 | 0.03 |
| Accidents (in- or outpatient) | 1.74 | 0.06 |  | 0.01 | 1.88 | 0.05 | 0.07 |  | 0.07 | 2.54 | 0.05 | <.0001 |
| Total Drug Costs | 167.39 | 2.62 |  | 0.07 | 202.41 | 2.58 | <.0001 |  | 0.30 | 328.64 | 2.54 | <.0001 |
| Duration of follow up (<5 years) |  |  |  |  |  |  |  |  |  |  |  |  |
| Frequency of Care |  |  |  |  |  |  |  |  |  |  |  |  |
| Outpatient Care^3^ | 84.39 | 0.67 |  | 0.05 | 91.72 | 0.69 | <.0001 |  | 0.15 | 109.88 | 0.93 | <.0001 |
| Inpatient Care^4^ | 2.43 | 0.03 |  | 0.02 | 2.54 | 0.03 | 0.02 |  | 0.03 | 2.64 | 0.04 | 0.0001 |
| Accidents (in- or outpatient) | 3.08 | 0.05 |  | 0.02 | 3.24 | 0.05 | 0.02 |  | 0.05 | 3.67 | 0.06 | <.0001 |
| Hospitalization Days^5^ | 33.31 | 0.82 |  | 0.03 | 37.84 | 0.84 | 0.0001 |  | 0.02 | 36.59 | 1.14 | 0.02 |
| Cost of Care^6^ (NT$/10^3^) |  |  |  |  |  |  |  |  |  |  |  |  |
| Outpatient Care^3^ | 181.80 | 2.76 |  | 0.04 | 202.31 | 2.81 | <.0001 |  | 0.10 | 252.68 | 3.82 | <.0001 |
| Inpatient Care^4^ | 222.89 | 4.57 |  | 0.01 | 236.59 | 4.66 | 0.04 |  | 0.01 | 235.02 | 6.34 | 0.12 |
| Accidents (in- or outpatient) | 14.59 | 0.26 |  | 0.02 | 15.90 | 0.26 | 0.0004 |  | 0.06 | 18.66 | 0.36 | <.0001 |
| Total Medical Costs | 419.28 | 5.67 |  | 0.03 | 454.80 | 5.78 | <.0001 |  | 0.06 | 506.37 | 7.87 | <.0001 |
| Drug cost (NT$/10^3^) |  |  |  |  |  |  |  |  |  |  |  |  |
| Outpatient Care^3^ | 78.95 | 1.42 |  | 0.07 | 98.28 | 1.44 | <.0001 |  | 0.16 | 136.14 | 1.96 | <.0001 |
| Inpatient Care^4^ | 34.49 | 0.81 |  | 0.01 | 35.74 | 0.82 | 0.28 |  | −0.002 | 34.17 | 1.12 | 0.82 |
| Accidents (in- or outpatient) | 1.24 | 0.04 |  | 0.02 | 1.39 | 0.04 | 0.01 |  | 0.05 | 1.73 | 0.06 | <.0001 |
| Total Drug Costs | 114.69 | 1.68 |  | 0.06 | 135.41 | 1.71 | <.0001 |  | 0.14 | 172.05 | 2.33 | <.0001 |
| Duration of follow up (5-10 years) |  |  |  |  |  |  |  |  |  |  |  |  |
| Frequency of Care |  |  |  |  |  |  |  |  |  |  |  |  |
| Outpatient Care^3^ | 202.49 | 1.80 |  | −0.01 | 199.95 | 1.69 | 0.30 |  | 0.02 | 208.10 | 1.39 | 0.01 |
| Inpatient Care^4^ | 3.86 | 0.06 |  | −0.005 | 3.81 | 0.06 | 0.57 |  | −0.02 | 3.71 | 0.05 | 0.07 |
| Accidents (in- or outpatient) | 5.38 | 0.10 |  | 0.01 | 5.55 | 0.10 | 0.23 |  | 0.02 | 5.73 | 0.08 | 0.01 |
| Hospitalization Days^5^ | 54.05 | 2.14 |  | 0.01 | 56.00 | 2.01 | 0.51 |  | −0.01 | 49.75 | 1.65 | 0.11 |
| Cost of Care^6^ (NT$/10^3^) |  |  |  |  |  |  |  |  |  |  |  |  |
| Outpatient Care^3^ | 389.30 | 6.54 |  | 0.02 | 415.29 | 6.15 | 0.004 |  | 0.09 | 473.69 | 5.04 | <.0001 |
| Inpatient Care^4^ | 316.11 | 9.44 |  | −0.003 | 311.88 | 8.87 | 0.74 |  | −0.01 | 303.33 | 7.28 | 0.29 |
| Accidents (in- or outpatient) | 23.69 | 0.52 |  | 0.01 | 24.66 | 0.49 | 0.17 |  | 0.03 | 25.68 | 0.40 | 0.003 |
| Total Medical Costs | 729.10 | 12.26 |  | 0.01 | 751.83 | 11.52 | 0.18 |  | 0.04 | 802.69 | 9.45 | <.0001 |
| Drug cost (NT$/10^3^) |  |  |  |  |  |  |  |  |  |  |  |  |
| Outpatient Care^3^ | 173.98 | 3.00 |  | 0.07 | 208.97 | 2.82 | <.0001 |  | 0.24 | 278.27 | 2.31 | <.0001 |
| Inpatient Care^4^ | 40.49 | 1.33 |  | −0.004 | 39.76 | 1.25 | 0.69 |  | −0.01 | 39.19 | 1.03 | 0.44 |
| Accidents (in- or outpatient) | 1.90 | 0.07 |  | 0.01 | 1.99 | 0.06 | 0.36 |  | 0.03 | 2.16 | 0.05 | 0.004 |
| Total Drug Costs | 216.38 | 3.39 |  | 0.06 | 250.72 | 3.18 | <.0001 |  | 0.21 | 319.62 | 2.61 | <.0001 |
| Duration of follow up (>10 years) |  |  |  |  |  |  |  |  |  |  |  |  |
| Frequency of Care |  |  |  |  |  |  |  |  |  |  |  |  |
| Outpatient Care^3^ | 351.71 | 4.53 |  | −0.002 | 350.93 | 4.83 | 0.91 |  | −0.07 | 324.47 | 3.29 | <.0001 |
| Inpatient Care^4^ | 4.52 | 0.11 |  | 0.001 | 4.53 | 0.12 | 0.93 |  | −0.01 | 4.38 | 0.08 | 0.32 |
| Accidents (in- or outpatient) | 7.53 | 0.22 |  | −0.002 | 7.49 | 0.24 | 0.88 |  | −0.003 | 7.48 | 0.16 | 0.85 |
| Hospitalization Days^5^ | 57.53 | 4.52 |  | 0.03 | 72.68 | 4.81 | 0.02 |  | −0.01 | 51.81 | 3.28 | 0.31 |
| Cost of Care^6^ (NT$/10^3^) |  |  |  |  |  |  |  |  |  |  |  |  |
| Outpatient Care^3^ | 600.25 | 13.69 |  | 0.02 | 630.51 | 14.57 | 0.13 |  | 0.12 | 747.64 | 9.95 | <.0001 |
| Inpatient Care^4^ | 322.78 | 17.88 |  | 0.02 | 354.74 | 19.03 | 0.22 |  | 0.0003 | 323.30 | 12.99 | 0.98 |
| Accidents (in- or outpatient) | 30.56 | 1.01 |  | −0.002 | 30.34 | 1.07 | 0.88 |  | 0.01 | 31.69 | 0.73 | 0.37 |
| Total Medical Costs | 953.60 | 23.99 |  | 0.02 | 1,015.59 | 25.54 | 0.08 |  | 0.07 | 1,102.62 | 17.44 | <.0001 |
| Drug cost (NT$/10^3^) |  |  |  |  |  |  |  |  |  |  |  |  |
| Outpatient Care^3^ | 262.43 | 5.97 |  | 0.10 | 332.22 | 6.36 | <.0001 |  | 0.38 | 482.50 | 4.34 | <.0001 |
| Inpatient Care^4^ | 37.37 | 2.02 |  | −0.002 | 36.99 | 2.15 | 0.90 |  | 0.01 | 38.44 | 1.47 | 0.67 |
| Accidents (in- or outpatient) | 2.27 | 0.12 |  | 0.0003 | 2.27 | 0.13 | 0.98 |  | 0.03 | 2.59 | 0.09 | 0.03 |
| Total Drug Costs | 302.07 | 6.51 |  | 0.09 | 371.48 | 6.93 | <.0001 |  | 0.36 | 523.54 | 4.73 | <.0001 |

SE, standard error. Standardized betas allow to compare differences between PD and non-PD subjects for the different costs. ^1^ Models were adjusted for age, gender, urbanization status, insurance premium, and comorbidity. 2 The reference group is Mild PD cases group. 3 Exclude accidents and emergency care/medical costs. 4 Exclude accidents care/medical costs. 5 Total hospitalization days including hospitalization due to accidents. 6 Costs were deflated by the consumer price index (CPI) with the base year of 2016.

**Appendix Table 3** Sociodemographic Characteristics of Deceased and Survived PD Participants

| Variables | Deceased  (N=20,466) | Survived  (N=29,824) | *p-value*^1^ |
| --- | --- | --- | --- |
|  | n (%) | n (%) |  |
| Age (years) |  |  | <.0001 |
| <70 | 3,329(16.27) | 13,597(45.59) |  |
| 70-74 | 3,694(18.05) | 5,967(20.01) |  |
| 75-79 | 5,511(26.93) | 5,491(18.41) |  |
| ≧80 | 7,932(38.76) | 4,769(15.99) |  |
| Mean (SD)^2^ | 76.72(8.04) | 69.49(10.25) |  |
| Gender |  |  | <.0001 |
| Female | 8,464(41.36) | 15,972(53.55) |  |
| Male | 12,002(58.64) | 13,852(46.45) |  |
| Insurance premium (NT$)^2^ |  |  | <.0001 |
| Dependent | 13,216(64.58) | 18,448(61.86) |  |
| <Median (19,200) | 2,459(12.02) | 3,313(11.11) |  |
| ≧Median | 4,791(23.41) | 8,063(27.04) |  |
| Mean (SD) | 13,000.46(12,441.50) | 21,152.44(20,888.12) |  |
| Urbanization status |  |  | <.0001 |
| Urban | 10,722(52.39) | 16,126(54.07) |  |
| Satellite city/town | 6,951(33.96) | 9,581(32.13) |  |
| Rural area | 2,793(13.65) | 4,117(13.80) |  |
| Charlson Comorbidity Index^3^ |  |  | <.0001 |
| 0 | 8,572(41.88) | 17,346(58.16) |  |
| 1 | 5,789(28.29) | 7,513(25.19) |  |
| ≥2 | 6,105(29.83) | 4,965(16.65) |  |

SD, Standard Deviation. ^1^ χ2 test. ^2^ NT$=New Taiwan Dollars. ^3^ Within one year before the index date.

**Appendix Table 4** Mean Predicted Values^1^ of Medical Care Utilization and Costs Among Patients with PD Stratified by Survival Status^2^

|  |  |  |  | Deceased  (N=20,466) | | Survived  (N=29,824) | | *p-value*^1^ |
| --- | --- | --- | --- | --- | --- | --- | --- | --- |
|  | Beta | SE | Standardized beta | predicted mean | SE | predicted mean | SE |  |
| Total |  |  |  |  |  |  |  |  |
| Frequency of Care |  |  |  |  |  |  |  |  |
| Outpatient Care^3^ | −24.28 | 1.45 | −0.08 | 156.13 | 1.08 | 180.41 | 0.88 | <.001 |
| Inpatient Care^4^ | 2.66 | 0.04 | 0.31 | 4.85 | 0.03 | 2.19 | 0.02 | <.001 |
| Accidents (in- or outpatient) | 2.14 | 0.07 | 0.15 | 6.03 | 0.05 | 3.89 | 0.04 | <.001 |
| Hospitalization Days^5^ | 53.46 | 1.28 | 0.20 | 77.11 | 0.96 | 23.65 | 0.78 | <.001 |
| Cost of Care^6^ (NT$/10^3^) |  |  |  |  |  |  |  |  |
| Outpatient Care^3^ | 11.50 | 4.39 | 0.01 | 367.72 | 3.27 | 356.22 | 2.67 | 0.01 |
| Inpatient Care^4^ | 323.08 | 5.69 | 0.26 | 466.39 | 4.25 | 143.32 | 3.47 | <.001 |
| Accidents (in- or outpatient) | 13.87 | 0.33 | 0.20 | 29.74 | 0.25 | 15.87 | 0.20 | <.001 |
| Total Medical Costs | 348.46 | 7.77 | 0.21 | 863.85 | 5.80 | 515.40 | 4.73 | <.001 |
| Drug cost (NT$/10^3^) |  |  |  |  |  |  |  |  |
| Outpatient Care^3^ | −17.51 | 2.16 | −0.04 | 180.37 | 1.61 | 197.89 | 1.31 | <.001 |
| Inpatient Care^4^ | 55.28 | 0.82 | 0.31 | 69.91 | 0.61 | 14.63 | 0.50 | <.001 |
| Accidents (in- or outpatient) | 1.65 | 0.04 | 0.17 | 2.77 | 0.03 | 1.12 | 0.03 | <.001 |
| Total Drug Costs | 39.42 | 2.39 | 0.08 | 253.05 | 1.79 | 213.64 | 1.46 | <.001 |
| Age (<72) |  |  |  |  |  |  |  |  |
| Frequency of Care |  |  |  |  |  |  |  |  |
| Outpatient Care^3^ | −12.07 | 2.67 | −0.03 | 168.97 | 2.34 | 181.04 | 1.23 | <.0001 |
| Inpatient Care^4^ | 3.15 | 0.06 | 0.33 | 5.12 | 0.06 | 1.98 | 0.03 | <.0001 |
| Accidents (in- or outpatient) | 2.81 | 0.12 | 0.17 | 6.12 | 0.10 | 3.32 | 0.05 | <.0001 |
| Hospitalization Days^5^ | 58.23 | 2.42 | 0.17 | 81.27 | 2.12 | 23.03 | 1.12 | <.0001 |
| Cost of Care^6^ (NT$/10^3^) |  |  |  |  |  |  |  |  |
| Outpatient Care^3^ | 71.73 | 9.24 | 0.05 | 478.03 | 8.10 | 406.30 | 4.26 | <.0001 |
| Inpatient Care^4^ | 367.84 | 9.38 | 0.27 | 506.39 | 8.23 | 138.55 | 4.33 | <.0001 |
| Accidents (in- or outpatient) | 19.09 | 0.56 | 0.24 | 31.64 | 0.49 | 12.55 | 0.26 | <.0001 |
| Total Medical Costs | 458.66 | 14.43 | 0.22 | 1016.06 | 12.66 | 557.40 | 6.65 | <.0001 |
| Drug cost (NT$/10^3^) |  |  |  |  |  |  |  |  |
| Outpatient Care^3^ | −12.79 | 4.56 | −0.02 | 219.91 | 4.00 | 232.70 | 2.10 | 0.01 |
| Inpatient Care^4^ | 64.06 | 1.46 | 0.30 | 77.25 | 1.28 | 13.18 | 0.67 | <.0001 |
| Accidents (in- or outpatient) | 2.06 | 0.07 | 0.22 | 2.90 | 0.06 | 0.83 | 0.03 | <.0001 |
| Total Drug Costs | 53.34 | 4.95 | 0.08 | 300.05 | 4.34 | 246.72 | 2.28 | <.0001 |
| Age (≧72) |  |  |  |  |  |  |  |  |
| Frequency of Care |  |  |  |  |  |  |  |  |
| Outpatient Care^3^ | −33.12 | 1.67 | −0.11 | 149.74 | 1.13 | 182.87 | 1.21 | <.0001 |
| Inpatient Care^4^ | 2.38 | 0.05 | 0.28 | 4.79 | 0.03 | 2.42 | 0.03 | <.0001 |
| Accidents (in- or outpatient) | 1.80 | 0.08 | 0.13 | 6.15 | 0.05 | 4.35 | 0.06 | <.0001 |
| Hospitalization Days^5^ | 50.01 | 1.45 | 0.20 | 75.20 | 0.99 | 25.19 | 1.05 | <.0001 |
| Cost of Care^6^ (NT$/10^3^) |  |  |  |  |  |  |  |  |
| Outpatient Care^3^ | −40.06 | 4.40 | −0.05 | 300.94 | 2.99 | 341.01 | 3.19 | <.0001 |
| Inpatient Care^4^ | 294.21 | 7.07 | 0.24 | 449.40 | 4.80 | 155.19 | 5.11 | <.0001 |
| Accidents (in- or outpatient) | 11.38 | 0.40 | 0.16 | 30.02 | 0.27 | 18.64 | 0.29 | <.0001 |
| Total Medical Costs | 265.53 | 8.90 | 0.17 | 780.37 | 6.04 | 514.84 | 6.44 | <.0001 |
| Drug cost (NT$/10^3^) |  |  |  |  |  |  |  |  |
| Outpatient Care^3^ | −33.00 | 2.19 | −0.09 | 149.03 | 1.49 | 182.03 | 1.59 | <.0001 |
| Inpatient Care^4^ | 49.91 | 0.96 | 0.29 | 67.06 | 0.65 | 17.15 | 0.69 | <.0001 |
| Accidents (in- or outpatient) | 1.44 | 0.06 | 0.14 | 2.80 | 0.04 | 1.36 | 0.04 | <.0001 |
| Total Drug Costs | 18.35 | 2.49 | 0.04 | 218.89 | 1.69 | 200.54 | 1.80 | <.0001 |
| Gender (Female) |  |  |  |  |  |  |  |  |
| Frequency of Care |  |  |  |  |  |  |  |  |
| Outpatient Care^3^ | −25.37 | 2.16 | −0.08 | 164.63 | 1.71 | 190.01 | 1.22 | <.0001 |
| Inpatient Care^4^ | 2.70 | 0.05 | 0.33 | 4.79 | 0.04 | 2.09 | 0.03 | <.0001 |
| Accidents (in- or outpatient) | 2.05 | 0.09 | 0.15 | 5.89 | 0.07 | 3.84 | 0.05 | <.0001 |
| Hospitalization Days^5^ | 52.43 | 1.79 | 0.20 | 74.29 | 1.41 | 21.86 | 1.01 | <.0001 |
| Cost of Care^6^ (NT$/10^3^) |  |  |  |  |  |  |  |  |
| Outpatient Care^3^ | 29.69 | 6.64 | 0.03 | 391.66 | 5.25 | 361.96 | 3.74 | <.0001 |
| Inpatient Care^4^ | 312.81 | 8.01 | 0.26 | 447.79 | 6.33 | 134.98 | 4.51 | <.0001 |
| Accidents (in- or outpatient) | 14.23 | 0.48 | 0.20 | 29.35 | 0.38 | 15.12 | 0.27 | <.0001 |
| Total Medical Costs | 356.73 | 11.29 | 0.21 | 868.80 | 8.92 | 512.07 | 6.36 | <.0001 |
| Drug cost (NT$/10^3^) |  |  |  |  |  |  |  |  |
| Outpatient Care^3^ | −9.42 | 3.14 | −0.02 | 186.66 | 2.48 | 196.08 | 1.77 | 0.003 |
| Inpatient Care^4^ | 50.92 | 1.04 | 0.32 | 63.10 | 0.82 | 12.18 | 0.59 | <.0001 |
| Accidents (in- or outpatient) | 1.52 | 0.06 | 0.18 | 2.50 | 0.05 | 0.98 | 0.03 | <.0001 |
| Total Drug Costs | 43.02 | 3.42 | 0.08 | 252.25 | 2.70 | 209.23 | 1.93 | <.0001 |
| Gender (Male) |  |  |  |  |  |  |  |  |
| Frequency of Care |  |  |  |  |  |  |  |  |
| Outpatient Care^3^ | −23.50 | 1.96 | −0.08 | 147.84 | 1.38 | 171.34 | 1.28 | <.0001 |
| Inpatient Care^4^ | 2.62 | 0.06 | 0.30 | 4.92 | 0.04 | 2.30 | 0.04 | <.0001 |
| Accidents (in- or outpatient) | 2.21 | 0.10 | 0.15 | 6.14 | 0.07 | 3.93 | 0.06 | <.0001 |
| Hospitalization Days^5^ | 54.40 | 1.84 | 0.20 | 79.64 | 1.30 | 25.24 | 1.20 | <.0001 |
| Cost of Care^6^ (NT$/10^3^) |  |  |  |  |  |  |  |  |
| Outpatient Care^3^ | −5.29 | 5.80 | −0.01 | 347.33 | 4.09 | 352.63 | 3.79 | 0.36 |
| Inpatient Care^4^ | 332.22 | 8.08 | 0.27 | 482.53 | 5.70 | 150.31 | 5.28 | <.0001 |
| Accidents (in- or outpatient) | 13.51 | 0.46 | 0.19 | 30.13 | 0.32 | 16.62 | 0.30 | <.0001 |
| Total Medical Costs | 340.44 | 10.72 | 0.21 | 860.00 | 7.56 | 519.56 | 7.00 | <.0001 |
| Drug cost (NT$/10^3^) |  |  |  |  |  |  |  |  |
| Outpatient Care^3^ | −24.57 | 2.98 | −0.05 | 175.65 | 2.10 | 200.22 | 1.94 | <.0001 |
| Inpatient Care^4^ | 59.06 | 1.24 | 0.30 | 75.69 | 0.88 | 16.62 | 0.81 | <.0001 |
| Accidents (in- or outpatient) | 1.76 | 0.07 | 0.17 | 3.01 | 0.05 | 1.25 | 0.04 | <.0001 |
| Total Drug Costs | 36.25 | 3.35 | 0.07 | 254.34 | 2.37 | 218.09 | 2.19 | <.0001 |
| Duration of follow up (<5 years) |  |  |  |  |  |  |  |  |
| Frequency of Care |  |  |  |  |  |  |  |  |
| Outpatient Care^3^ | −13.39 | 0.93 | −0.10 | 85.63 | 0.65 | 99.02 | 0.62 | <.0001 |
| Inpatient Care^4^ | 2.45 | 0.04 | 0.38 | 3.79 | 0.03 | 1.34 | 0.03 | <.0001 |
| Accidents (in- or outpatient) | 2.01 | 0.06 | 0.22 | 4.31 | 0.04 | 2.30 | 0.04 | <.0001 |
| Hospitalization Days^5^ | 45.99 | 1.09 | 0.28 | 59.61 | 0.76 | 13.62 | 0.73 | <.0001 |
| Cost of Care^6^ (NT$/10^3^) |  |  |  |  |  |  |  |  |
| Outpatient Care^3^ | 11.32 | 3.82 | 0.02 | 210.49 | 2.65 | 199.17 | 2.54 | 0.003 |
| Inpatient Care^4^ | 301.45 | 6.00 | 0.33 | 387.16 | 4.16 | 85.71 | 3.99 | <.0001 |
| Accidents (in- or outpatient) | 12.79 | 0.35 | 0.24 | 22.59 | 0.24 | 9.80 | 0.23 | <.0001 |
| Total Medical Costs | 325.57 | 7.55 | 0.28 | 620.25 | 5.23 | 294.68 | 5.02 | <.0001 |
| Drug cost (NT$/10^3^) |  |  |  |  |  |  |  |  |
| Outpatient Care^3^ | −10.44 | 1.97 | −0.04 | 93.02 | 1.37 | 103.46 | 1.31 | <.0001 |
| Inpatient Care^4^ | 54.44 | 1.06 | 0.34 | 63.16 | 0.73 | 8.72 | 0.70 | <.0001 |
| Accidents (in- or outpatient) | 1.52 | 0.06 | 0.18 | 2.19 | 0.04 | 0.67 | 0.04 | <.0001 |
| Total Drug Costs | 45.52 | 2.32 | 0.13 | 158.37 | 1.61 | 112.85 | 1.54 | <.0001 |
| Duration of follow up (5-10 years) |  |  |  |  |  |  |  |  |
| Frequency of Care |  |  |  |  |  |  |  |  |
| Outpatient Care^3^ | 9.35 | 2.03 | 0.03 | 210.06 | 1.56 | 200.71 | 1.19 | <.0001 |
| Inpatient Care^4^ | 3.46 | 0.07 | 0.36 | 5.94 | 0.05 | 2.49 | 0.04 | <.0001 |
| Accidents (in- or outpatient) | 3.19 | 0.11 | 0.21 | 7.58 | 0.09 | 4.39 | 0.07 | <.0001 |
| Hospitalization Days^5^ | 70.32 | 2.36 | 0.23 | 96.67 | 1.82 | 26.35 | 1.39 | <.0001 |
| Cost of Care^6^ (NT$/10^3^) |  |  |  |  |  |  |  |  |
| Outpatient Care^3^ | 85.82 | 7.38 | 0.09 | 487.91 | 5.69 | 402.09 | 4.34 | <.0001 |
| Inpatient Care^4^ | 393.38 | 10.26 | 0.28 | 555.03 | 7.91 | 161.65 | 6.03 | <.0001 |
| Accidents (in- or outpatient) | 18.25 | 0.57 | 0.23 | 36.26 | 0.44 | 18.01 | 0.34 | <.0001 |
| Total Medical Costs | 497.45 | 13.37 | 0.27 | 1079.20 | 10.31 | 581.75 | 7.85 | <.0001 |
| Drug cost (NT$/10^3^) |  |  |  |  |  |  |  |  |
| Outpatient Care^3^ | 12.89 | 3.45 | 0.03 | 238.48 | 2.66 | 225.58 | 2.03 | 0.0002 |
| Inpatient Care^4^ | 61.18 | 1.44 | 0.31 | 77.93 | 1.11 | 16.75 | 0.84 | <.0001 |
| Accidents (in- or outpatient) | 2.02 | 0.08 | 0.20 | 3.31 | 0.06 | 1.28 | 0.04 | <.0001 |
| Total Drug Costs | 76.10 | 3.85 | 0.15 | 319.71 | 2.97 | 243.61 | 2.26 | <.0001 |
| Duration of follow up (>10 years) |  |  |  |  |  |  |  |  |
| Frequency of Care |  |  |  |  |  |  |  |  |
| Outpatient Care^3^ | −15.28 | 5.67 | −0.03 | 326.32 | 4.85 | 341.59 | 2.71 | 0.01 |
| Inpatient Care^4^ | 3.01 | 0.13 | 0.26 | 6.72 | 0.11 | 3.71 | 0.06 | <.0001 |
| Accidents (in- or outpatient) | 3.01 | 0.28 | 0.13 | 9.76 | 0.24 | 6.75 | 0.13 | <.0001 |
| Hospitalization Days^5^ | 57.49 | 5.60 | 0.12 | 101.44 | 4.80 | 43.96 | 2.68 | <.0001 |
| Cost of Care^6^ (NT$/10^3^) |  |  |  |  |  |  |  |  |
| Outpatient Care^3^ | 77.72 | 17.15 | 0.05 | 739.99 | 14.70 | 662.27 | 8.21 | <.0001 |
| Inpatient Care^4^ | 324.30 | 21.99 | 0.18 | 574.55 | 18.84 | 250.25 | 10.52 | <.0001 |
| Accidents (in- or outpatient) | 19.54 | 1.24 | 0.18 | 45.78 | 1.06 | 26.24 | 0.59 | <.0001 |
| Total Medical Costs | 421.56 | 29.58 | 0.17 | 1360.32 | 25.34 | 938.76 | 14.15 | <.0001 |
| Drug cost (NT$/10^3^) |  |  |  |  |  |  |  |  |
| Outpatient Care^3^ | 16.11 | 7.93 | 0.02 | 401.60 | 6.79 | 385.49 | 3.79 | 0.04 |
| Inpatient Care^4^ | 52.96 | 2.45 | 0.25 | 77.68 | 2.10 | 24.72 | 1.17 | <.0001 |
| Accidents (in- or outpatient) | 2.19 | 0.15 | 0.17 | 4.08 | 0.13 | 1.89 | 0.07 | <.0001 |
| Total Drug Costs | 71.26 | 8.53 | 0.10 | 483.36 | 7.31 | 412.10 | 4.08 | <.0001 |

SE, standard error. Betas represent the difference in costs between PD and non-PD subjects. Standardized betas allow to compare differences for the different costs. ^1^ Models were adjusted for age, gender, urbanization status, insurance premium, and comorbidity. ^2^ The average length of follow-up was 4.97 years for deceased cases and 6.57 years for survived cases. The total length of follow-up was 101,817.57 years for deceased cases and 195,893.62 years for survived cases. ^3^ Exclude accidents and emergency care/medical costs. ^4^ Exclude accidents care/medical cost. ^5^ Total hospitalization days including hospitalization due to accidents. ^6^ Costs were deflated by the consumer price index (CPI) with the base year of 2016.

**Appendix Table 5** Mean Predicted Values^1^ of Medical Care Utilization and Costs Among Deceased Cases, by Severity of PD^2^

|  | Mild PD cases (N=7,679) | |  | Moderate PD cases (N=7,032) | | | | |  | Severe PD cases (N=5,755) | | |  |
| --- | --- | --- | --- | --- | --- | --- | --- | --- | --- | --- | --- | --- | --- |
|  | Predicted mean | SE |  | Standardized betas^3^ | Predicted mean | SE | | *p-value*^1^ |  | Standardized  betas^3^ | Predicted mean | SE | *p-value*^1^ |
| Total |  |  |  |  |  |  | |  |  |  |  |  |  |
| Frequency of Care |  |  |  |  |  |  | |  |  |  |  |  |  |
| Outpatient Care^4^ | 134.11 | 1.57 |  | 0.04 | 145.06 | 1.64 | | <.0001 |  | 0.21 | 199.97 | 1.82 | <.0001 |
| Inpatient Care^5^ | 4.63 | 0.05 |  | 0.03 | 4.95 | 0.06 | | <.0001 |  | 0.07 | 5.44 | 0.06 | <.0001 |
| Accidents (in- or outpatient) | 5.57 | 0.09 |  | 0.03 | 6.09 | 0.09 | | <.0001 |  | 0.11 | 7.49 | 0.10 | <.0001 |
| Hospitalization Days^6^ | 69.53 | 1.79 |  | 0.04 | 81.65 | 1.87 | | <.0001 |  | 0.04 | 84.77 | 2.08 | <.0001 |
| Cost of Care^7^ (NT$/10^3^) |  |  |  |  |  |  | |  |  |  |  |  |  |
| Outpatient Care^4^ | 293.84 | 5.63 |  | 0.03 | 325.74 | 5.86 | | <.0001 |  | 0.15 | 463.69 | 6.53 | <.0001 |
| Inpatient Care^5^ | 433.04 | 8.80 |  | 0.03 | 479.90 | 9.17 | | 0.0002 |  | 0.04 | 509.14 | 10.21 | <.0001 |
| Accidents (in- or outpatient) | 27.32 | 0.47 |  | 0.04 | 30.44 | 0.49 | | <.0001 |  | 0.11 | 37.07 | 0.55 | <.0001 |
| Total Medical Costs | 754.20 | 11.05 |  | 0.04 | 836.08 | 11.52 | | <.0001 |  | 0.12 | 1009.90 | 12.82 | <.0001 |
| Drug cost (NT$/10^3^) |  |  |  |  |  |  | |  |  |  |  |  |  |
| Outpatient Care^4^ | 115.17 | 2.30 |  | 0.07 | 147.32 | 2.40 | | <.0001 |  | 0.30 | 259.38 | 2.67 | <.0001 |
| Inpatient Care^5^ | 66.82 | 1.27 |  | 0.02 | 71.86 | 1.33 | | 0.01 |  | 0.03 | 74.78 | 1.48 | <.0001 |
| Accidents (in- or outpatient) | 2.46 | 0.07 |  | 0.03 | 2.83 | 0.07 | | 0.0002 |  | 0.08 | 3.60 | 0.08 | <.0001 |
| Total Drug Costs | 184.45 | 2.73 |  | 0.07 | 222.00 | 2.84 | | <.0001 |  | 0.27 | 337.76 | 3.16 | <.0001 |
| Age(<72) |  |  |  |  |  | |  |  |  |  |  |  |  |
| Frequency of Care |  |  |  |  |  | |  |  |  |  |  |  |  |
| Outpatient Care^3^ | 150.14 | 3.94 |  | 0.02 | 157.44 | | 4.10 | 0.20 |  | 0.20 | 215.46 | 3.94 | <.0001 |
| Inpatient Care^4^ | 5.11 | 0.14 |  | 0.01 | 5.18 | | 0.14 | 0.72 |  | 0.05 | 5.66 | 0.14 | 0.01 |
| Accidents (in- or outpatient) | 5.91 | 0.21 |  | −0.002 | 5.87 | | 0.22 | 0.89 |  | 0.08 | 7.32 | 0.21 | <.0001 |
| Hospitalization Days^5^ | 71.80 | 4.52 |  | 0.03 | 84.15 | | 4.70 | 0.06 |  | 0.06 | 93.88 | 4.51 | 0.001 |
| Cost of Care^6^ (NT$/10^3^) |  |  |  |  |  | |  |  |  |  |  |  |  |
| Outpatient Care^3^ | 439.05 | 18.91 |  | 0.02 | 467.04 | | 19.67 | 0.30 |  | 0.12 | 634.90 | 18.89 | <.0001 |
| Inpatient Care^4^ | 474.12 | 22.00 |  | 0.02 | 510.02 | | 22.88 | 0.26 |  | 0.06 | 575.70 | 21.97 | 0.001 |
| Accidents (in- or outpatient) | 29.31 | 1.33 |  | 0.03 | 33.06 | | 1.38 | 0.05 |  | 0.07 | 36.97 | 1.32 | <.0001 |
| Total Medical Costs | 942.48 | 31.27 |  | 0.03 | 1010.12 | | 32.53 | 0.13 |  | 0.12 | 1247.57 | 31.24 | <.0001 |
| Drug cost (NT$/10^3^) |  |  |  |  |  | |  |  |  |  |  |  |  |
| Outpatient Care^3^ | 144.51 | 6.83 |  | 0.07 | 186.27 | | 7.11 | <.0001 |  | 0.33 | 338.02 | 6.83 | <.0001 |
| Inpatient Care^4^ | 76.28 | 3.46 |  | 0.003 | 77.10 | | 3.60 | 0.87 |  | 0.03 | 84.53 | 3.46 | 0.09 |
| Accidents (in- or outpatient) | 2.58 | 0.16 |  | 0.03 | 2.96 | | 0.17 | 0.10 |  | 0.07 | 3.43 | 0.16 | 0.0002 |
| Total Drug Costs | 223.36 | 7.98 |  | 0.06 | 266.33 | | 8.30 | 0.0002 |  | 0.30 | 425.99 | 7.97 | <.0001 |
| Age(>=72) |  |  |  |  |  | |  |  |  |  |  |  |  |
| Frequency of Care |  |  |  |  |  | |  |  |  |  |  |  |  |
| Outpatient Care^3^ | 128.83 | 1.70 |  | 0.04 | 140.67 | | 1.77 | <.0001 |  | 0.22 | 197.90 | 2.05 | <.0001 |
| Inpatient Care^4^ | 4.48 | 0.06 |  | 0.04 | 4.86 | | 0.06 | <.0001 |  | 0.09 | 5.44 | 0.07 | <.0001 |
| Accidents (in- or outpatient) | 5.46 | 0.10 |  | 0.04 | 6.12 | | 0.10 | <.0001 |  | 0.12 | 7.60 | 0.12 | <.0001 |
| Hospitalization Days^5^ | 68.55 | 1.92 |  | 0.04 | 80.60 | | 2.01 | <.0001 |  | 0.04 | 82.59 | 2.32 | <.0001 |
| Cost of Care^6^ (NT$/10^3^) |  |  |  |  |  | |  |  |  |  |  |  |  |
| Outpatient Care^3^ | 247.07 | 5.09 |  | 0.04 | 282.35 | | 5.31 | <.0001 |  | 0.19 | 427.18 | 6.15 | <.0001 |
| Inpatient Care^4^ | 418.77 | 9.49 |  | 0.03 | 468.73 | | 9.90 | 0.0003 |  | 0.05 | 495.47 | 11.48 | <.0001 |
| Accidents (in- or outpatient) | 26.65 | 0.48 |  | 0.04 | 29.63 | | 0.50 | <.0001 |  | 0.13 | 37.57 | 0.58 | <.0001 |
| Total Medical Costs | 692.49 | 11.32 |  | 0.05 | 780.70 | | 11.81 | <.0001 |  | 0.13 | 960.23 | 13.69 | <.0001 |
| Drug cost (NT$/10^3^) |  |  |  |  |  | |  |  |  |  |  |  |  |
| Outpatient Care^3^ | 104.73 | 2.30 |  | 0.08 | 134.90 | | 2.40 | <.0001 |  | 0.31 | 238.66 | 2.78 | <.0001 |
| Inpatient Care^4^ | 63.79 | 1.33 |  | 0.03 | 69.92 | | 1.38 | 0.001 |  | 0.04 | 73.04 | 1.60 | <.0001 |
| Accidents (in- or outpatient) | 2.42 | 0.08 |  | 0.03 | 2.78 | | 0.08 | 0.001 |  | 0.09 | 3.68 | 0.09 | <.0001 |
| Total Drug Costs | 170.94 | 2.76 |  | 0.08 | 207.60 | | 2.87 | <.0001 |  | 0.28 | 315.39 | 3.33 | <.0001 |
| Gender (Female) |  |  |  |  |  | |  |  |  |  |  |  |  |
| Frequency of Care |  |  |  |  |  | |  |  |  |  |  |  |  |
| Outpatient Care^3^ | 145.08 | 2.40 |  | 0.02 | 150.51 | | 2.55 | 0.12 |  | 0.18 | 203.63 | 2.89 | <.0001 |
| Inpatient Care^4^ | 4.61 | 0.08 |  | 0.02 | 4.80 | | 0.09 | 0.11 |  | 0.07 | 5.31 | 0.10 | <.0001 |
| Accidents (in- or outpatient) | 5.47 | 0.12 |  | 0.03 | 5.95 | | 0.13 | 0.01 |  | 0.11 | 7.19 | 0.15 | <.0001 |
| Hospitalization Days^5^ | 67.22 | 2.81 |  | 0.02 | 74.40 | | 2.99 | 0.08 |  | 0.05 | 84.23 | 3.38 | 0.0001 |
| Cost of Care^6^ (NT$/10^3^) |  |  |  |  |  | |  |  |  |  |  |  |  |
| Outpatient Care^3^ | 326.78 | 9.48 |  | 0.02 | 351.22 | | 10.08 | 0.08 |  | 0.12 | 484.53 | 11.41 | <.0001 |
| Inpatient Care^4^ | 416.07 | 13.76 |  | 0.01 | 438.61 | | 14.63 | 0.26 |  | 0.05 | 500.21 | 16.56 | 0.0001 |
| Accidents (in- or outpatient) | 27.33 | 0.77 |  | 0.03 | 30.18 | | 0.82 | 0.01 |  | 0.08 | 35.53 | 0.93 | <.0001 |
| Total Medical Costs | 770.18 | 17.70 |  | 0.02 | 820.01 | | 18.82 | 0.05 |  | 0.11 | 1020.27 | 21.31 | <.0001 |
| Drug cost (NT$/10^3^) |  |  |  |  |  | |  |  |  |  |  |  |  |
| Outpatient Care^3^ | 125.01 | 3.54 |  | 0.06 | 153.66 | | 3.76 | <.0001 |  | 0.28 | 262.19 | 4.26 | <.0001 |
| Inpatient Care^4^ | 60.50 | 1.89 |  | 0.01 | 62.11 | | 2.01 | 0.56 |  | 0.03 | 66.98 | 2.28 | 0.03 |
| Accidents (in- or outpatient) | 2.22 | 0.10 |  | 0.03 | 2.60 | | 0.10 | 0.01 |  | 0.06 | 3.03 | 0.12 | <.0001 |
| Total Drug Costs | 187.72 | 4.16 |  | 0.06 | 218.37 | | 4.42 | <.0001 |  | 0.25 | 332.20 | 5.00 | <.0001 |
| Gender (Male) |  |  |  |  |  | |  |  |  |  |  |  |  |
| Frequency of Care |  |  |  |  |  | |  |  |  |  |  |  |  |
| Outpatient Care^3^ | 126.75 | 2.07 |  | 0.05 | 141.15 | | 2.12 | <.0001 |  | 0.22 | 196.64 | 2.33 | <.0001 |
| Inpatient Care^4^ | 4.66 | 0.07 |  | 0.04 | 5.06 | | 0.08 | 0.0001 |  | 0.08 | 5.51 | 0.08 | <.0001 |
| Accidents (in- or outpatient) | 5.65 | 0.12 |  | 0.03 | 6.18 | | 0.12 | 0.002 |  | 0.11 | 7.68 | 0.14 | <.0001 |
| Hospitalization Days^5^ | 71.19 | 2.32 |  | 0.05 | 86.73 | | 2.38 | <.0001 |  | 0.04 | 85.18 | 2.62 | <.0001 |
| Cost of Care^6^ (NT$/10^3^) |  |  |  |  |  | |  |  |  |  |  |  |  |
| Outpatient Care^3^ | 271.19 | 6.84 |  | 0.04 | 307.26 | | 7.03 | 0.0002 |  | 0.17 | 448.29 | 7.73 | <.0001 |
| Inpatient Care^4^ | 445.06 | 11.44 |  | 0.04 | 508.83 | | 11.76 | 0.0001 |  | 0.04 | 515.72 | 12.93 | <.0001 |
| Accidents (in- or outpatient) | 27.34 | 0.59 |  | 0.04 | 30.61 | | 0.60 | 0.0001 |  | 0.12 | 38.07 | 0.66 | <.0001 |
| Total Medical Costs | 743.59 | 14.09 |  | 0.05 | 846.70 | | 14.48 | <.0001 |  | 0.12 | 1002.07 | 15.93 | <.0001 |
| Drug cost (NT$/10^3^) |  |  |  |  |  | |  |  |  |  |  |  |  |
| Outpatient Care^3^ | 108.29 | 3.03 |  | 0.08 | 142.87 | | 3.11 | <.0001 |  | 0.32 | 256.95 | 3.42 | <.0001 |
| Inpatient Care^4^ | 71.37 | 1.71 |  | 0.03 | 78.63 | | 1.76 | 0.003 |  | 0.04 | 80.21 | 1.94 | 0.001 |
| Accidents (in- or outpatient) | 2.63 | 0.09 |  | 0.03 | 2.99 | | 0.10 | 0.01 |  | 0.10 | 3.99 | 0.11 | <.0001 |
| Total Drug Costs | 182.30 | 3.61 |  | 0.08 | 224.49 | | 3.71 | <.0001 |  | 0.28 | 341.15 | 4.08 | <.0001 |
| Duration of follow up (<5) |  |  |  |  |  | |  |  |  |  |  |  |  |
| Frequency of Care |  |  |  |  |  | |  |  |  |  |  |  |  |
| Outpatient Care^3^ | 77.67 | 0.95 |  | 0.08 | 88.90 | | 1.02 | <.0001 |  | 0.20 | 114.96 | 1.55 | <.0001 |
| Inpatient Care^4^ | 3.62 | 0.05 |  | 0.05 | 4.01 | | 0.05 | <.0001 |  | 0.07 | 4.36 | 0.08 | <.0001 |
| Accidents (in- or outpatient) | 4.13 | 0.07 |  | 0.04 | 4.57 | | 0.08 | <.0001 |  | 0.10 | 5.52 | 0.12 | <.0001 |
| Hospitalization Days^5^ | 53.64 | 1.39 |  | 0.06 | 66.35 | | 1.49 | <.0001 |  | 0.05 | 68.10 | 2.26 | <.0001 |
| Cost of Care^6^ (NT$/10^3^) |  |  |  |  |  | |  |  |  |  |  |  |  |
| Outpatient Care^3^ | 186.43 | 4.35 |  | 0.04 | 214.21 | | 4.68 | <.0001 |  | 0.11 | 279.20 | 7.08 | <.0001 |
| Inpatient Care^4^ | 358.97 | 8.01 |  | 0.05 | 413.22 | | 8.62 | <.0001 |  | 0.05 | 438.60 | 13.03 | <.0001 |
| Accidents (in- or outpatient) | 20.84 | 0.42 |  | 0.05 | 24.09 | | 0.45 | <.0001 |  | 0.11 | 30.14 | 0.69 | <.0001 |
| Total Medical Costs | 566.24 | 9.53 |  | 0.06 | 651.52 | | 10.24 | <.0001 |  | 0.10 | 747.94 | 15.49 | <.0001 |
| Drug cost (NT$/10^3^) |  |  |  |  |  | |  |  |  |  |  |  |  |
| Outpatient Care^3^ | 72.37 | 1.94 |  | 0.08 | 95.34 | | 2.08 | <.0001 |  | 0.18 | 142.57 | 3.15 | <.0001 |
| Inpatient Care^4^ | 59.12 | 1.43 |  | 0.04 | 66.88 | | 1.54 | 0.0002 |  | 0.04 | 69.72 | 2.32 | 0.0001 |
| Accidents (in- or outpatient) | 1.93 | 0.07 |  | 0.04 | 2.35 | | 0.08 | 0.0001 |  | 0.08 | 3.11 | 0.12 | <.0001 |
| Total Drug Costs | 133.42 | 2.49 |  | 0.08 | 164.58 | | 2.68 | <.0001 |  | 0.17 | 215.40 | 4.05 | <.0001 |
| Duration of follow up (5-10) |  |  |  |  |  | |  |  |  |  |  |  |  |
| Frequency of Care |  |  |  |  |  | |  |  |  |  |  |  |  |
| Outpatient Care^3^ | 220.22 | 2.97 |  | −0.02 | 214.61 | | 2.87 | 0.17 |  | −0.003 | 219.40 | 2.48 | 0.83 |
| Inpatient Care^4^ | 6.45 | 0.12 |  | −0.01 | 6.36 | | 0.12 | 0.58 |  | −0.06 | 5.83 | 0.10 | 0.0001 |
| Accidents (in- or outpatient) | 7.97 | 0.19 |  | 0.01 | 8.25 | | 0.19 | 0.29 |  | 0.002 | 8.00 | 0.16 | 0.90 |
| Hospitalization Days^5^ | 101.01 | 4.40 |  | 0.01 | 105.01 | | 4.26 | 0.51 |  | −0.02 | 93.08 | 3.67 | 0.17 |
| Cost of Care^6^ (NT$/10^3^) |  |  |  |  |  | |  |  |  |  |  |  |  |
| Outpatient Care^3^ | 462.24 | 12.77 |  | 0.01 | 472.51 | | 12.36 | 0.563 |  | 0.04 | 508.68 | 10.65 | 0.01 |
| Inpatient Care^4^ | 579.30 | 20.61 |  | 0.003 | 584.45 | | 19.95 | 0.86 |  | −0.02 | 541.05 | 17.19 | 0.16 |
| Accidents (in- or outpatient) | 37.91 | 1.05 |  | 0.01 | 39.31 | | 1.02 | 0.33 |  | 0.01 | 38.56 | 0.88 | 0.63 |
| Total Medical Costs | 1079.44 | 25.05 |  | 0.01 | 1096.27 | | 24.25 | 0.63 |  | 0.004 | 1088.30 | 20.90 | 0.79 |
| Drug cost (NT$/10^3^) |  |  |  |  |  | |  |  |  |  |  |  |  |
| Outpatient Care^3^ | 182.80 | 4.93 |  | 0.06 | 213.57 | | 4.77 | <.0001 |  | 0.21 | 281.05 | 4.11 | <.0001 |
| Inpatient Care^4^ | 81.31 | 2.64 |  | 0.0002 | 81.38 | | 2.56 | 0.99 |  | −0.02 | 77.16 | 2.20 | 0.23 |
| Accidents (in- or outpatient) | 3.31 | 0.15 |  | 0.02 | 3.56 | | 0.14 | 0.22 |  | 0.02 | 3.65 | 0.12 | 0.081 |
| Total Drug Costs | 267.43 | 5.74 |  | 0.05 | 298.51 | | 5.56 | <.0001 |  | 0.18 | 361.86 | 4.79 | <.0001 |
| Duration of follow up(>10) |  |  |  |  |  | |  |  |  |  |  |  |  |
| Frequency of Care |  |  |  |  |  | |  |  |  |  |  |  |  |
| Outpatient Care^3^ | 354.78 | 8.84 |  | 0.02 | 362.68 | | 9.39 | 0.54 |  | −0.04 | 337.95 | 6.60 | 0.13 |
| Inpatient Care^4^ | 7.65 | 0.28 |  | −0.01 | 7.54 | | 0.30 | 0.79 |  | −0.08 | 6.60 | 0.21 | 0.003 |
| Accidents (in- or outpatient) | 10.46 | 0.53 |  | 0.02 | 10.92 | | 0.57 | 0.56 |  | −0.01 | 10.32 | 0.40 | 0.84 |
| Hospitalization Days^5^ | 106.00 | 10.77 |  | 0.03 | 122.92 | | 11.43 | 0.28 |  | −0.02 | 94.96 | 8.04 | 0.42 |
| Cost of Care^6^ (NT$/10^3^) |  |  |  |  |  | |  |  |  |  |  |  |  |
| Outpatient Care^3^ | 707.08 | 32.70 |  | 0.01 | 723.72 | | 34.71 | 0.73 |  | 0.02 | 740.15 | 24.42 | 0.42 |
| Inpatient Care^4^ | 603.66 | 48.45 |  | 0.02 | 644.82 | | 51.42 | 0.56 |  | −0.02 | 564.67 | 36.18 | 0.52 |
| Accidents (in- or outpatient) | 50.70 | 2.75 |  | 0.01 | 51.43 | | 2.92 | 0.86 |  | −0.02 | 48.03 | 2.05 | 0.44 |
| Total Medical Costs | 1361.44 | 61.79 |  | 0.02 | 1419.97 | | 65.58 | 0.52 |  | −0.003 | 1352.85 | 46.14 | 0.91 |
| Drug cost (NT$/10^3^) |  |  |  |  |  | |  |  |  |  |  |  |  |
| Outpatient Care^3^ | 268.93 | 12.20 |  | 0.10 | 340.37 | | 12.94 | <.0001 |  | 0.32 | 458.73 | 9.11 | <.0001 |
| Inpatient Care^4^ | 88.27 | 5.93 |  | −0.04 | 75.65 | | 6.29 | 0.14 |  | −0.04 | 78.23 | 4.43 | 0.18 |
| Accidents (in- or outpatient) | 4.50 | 0.33 |  | −0.02 | 4.09 | | 0.35 | 0.39 |  | −0.001 | 4.49 | 0.24 | 0.98 |
| Total Drug Costs | 361.69 | 14.05 |  | 0.07 | 420.11 | | 14.91 | 0.004 |  | 0.27 | 541.44 | 10.49 | <.0001 |

SE, standard error. Standardized betas allow to compare differences between PD and non-PD subjects for the different costs. ^1^  Models were adjusted for age, gender, urbanization status, insurance premium, and comorbidity. 2 The average length of follow-up was 4.15 years for Mild deceased-cases and 4.51 years for Moderate deceased-cases and 6.64 years for Sever deceased-cases cases. The total length of follow-up was 31,882.50 years for Mild deceased-cases cases and 31,701.69 years for Moderate deceased-cases cases and 38,233.38 years for Sever deceased-cases. 3 The reference group is Mild deceased-cases group. 4 Exclude accidents and emergency care/medical costs. 5 Exclude accidents care/medical costs. 6 Total hospitalization days including hospitalization due to accidents. 7 Costs were deflated by the consumer price index (CPI) with the base year of 2016.
